# Photochemistry of Ru^II^ 4,4′-Bi-1,2,3-triazolyl (btz) Complexes: Crystallographic Characterization of the Photoreactive Ligand-Loss Intermediate *trans*-[Ru(bpy)(κ^2^-btz)(κ^1^-btz)(NCMe)]^2+^

**DOI:** 10.1002/chem.201402354

**Published:** 2014-05-30

**Authors:** Christine E Welby, Georgina K Armitage, Harry Bartley, Aaron Wilkinson, Alessandro Sinopoli, Baljinder S Uppal, Craig R Rice, Paul I P Elliott

**Affiliations:** [a] Department of Chemistry, University of HuddersfieldQueensgate, Huddersfield, HD1 3DH (UK) E-mail: p.i.elliott@hud.ac.uk

**Keywords:** coordination chemistry, N ligands, photochemistry, ruthenium

## Abstract

We report the unprecedented observation and unequivocal crystallographic characterization of the *meta*-stable ligand loss intermediate solvento complex *trans*-[Ru(bpy)(κ^2^-btz)(κ^1^-btz)(NCMe)]^2+^ (**1 a**) that contains a monodentate chelate ligand. This and analogous complexes can be observed during the photolysis reactions of a family of complexes of the form [Ru(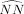)(btz)_2_]^2+^ (**1 a**–**d**: btz=1,1′-dibenzyl-4,4′-bi-1,2,3-triazolyl; 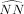
=a) 2,2′-bipyridyl (bpy), b) 4,4′-dimethyl-2,2′-bipyridyl (dmbpy), c) 4,4′-dimethoxy-2,2′-bipyridyl (dmeobpy), d) 1,10-phenanthroline (phen)). In acetonitrile solutions, **1 a**–**d** eventually convert to the bis-solvento complexes *trans*-[Ru(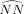)(btz)(NCMe)_2_]^2+^ (**3 a**–**d**) along with one equivalent of free btz, in a process in which the remaining coordinated bidentate ligands undergo a new rearrangement such that they become coplanar. X-ray crystal structure of **3 a** and **3 d** confirmed the co-planar arrangement of the 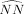 and btz ligands and the *trans* coordination of two solvent molecules. These conversions proceed via the observed intermediate complexes **2 a**–**d**, which are formed quantitatively from **1 a**–**d** in a matter of minutes and to which they slowly revert back on being left to stand in the dark over several days. The remarkably long lifetime of the intermediate complexes (>12 h at 40 °C) allowed the isolation of **2 a** in the solid state, and the complex to be crystallographically characterized. Similarly to the structures adopted by complexes **3 a** and **d**, the bpy and κ^2^-btz ligands in **2 a** coordinate in a square-planar fashion with the second monodentate btz ligand coordinated *trans* to an acetonitrile ligand.

## Introduction

The photophysics of ruthenium(II) polypyridyl complexes,[1] and other d^6^ complexes containing Os^II^, Re^I^ and Ir^III^, have been the subject of intense investigation over the past four decades. This interest stems from the potential applications of these complexes in artificial photosynthesis and light harvesting. The visible-absorption spectra of complexes, such as [Ru(bpy)_3_]^2+^, are dominated by metal–ligand charge transfer (^1^MLCT) transitions, which due to the presence of the heavy-metal atom undergo rapid intersystem crossing to lower energy ^3^MLCT states. These ^3^MLCT states are themselves photochemically inert but may, however, allow thermal population of photolabile ^3^MC states (characterized by population of the dσ* orbitals). The population of these ^3^MC states may result in dechelation of ligands generating vacant sites accessible to coordination by the solvent and ultimately isomerisation or ligand dissociation reactions.[2]

There has been renewed interest in the investigation of the photophysics of these complexes and the tuning of the relative energies of the ^3^MLCT and ^3^MC states.[3] By designing complexes, in which these states are brought into close proximity, the thermal population of ^3^MC from photoexcited ^3^MLCT states has been exploited for the light-activated formation of anti-cancer agents for photodynamic therapy.[3e, 4] In systems analogous to the classic complex [Ru(bpy)_3_]^2+^, inclusion of steric congestion (with substituents adjacent to the coordinating N-donor atoms of the ligand that is lost, for example) promotes photolability through stabilization of ^3^MC states relative to the ^3^MLCT state.

A key intermediate proposed in ligand loss and isomerisation reactions was long assumed to involve a complex of the form [Ru(κ^2^-bpy)_2_(κ^1^-bpy)(solvent)]^2+^.[5] Indeed, electrospray mass spectrometry and trace detection by ^1^H NMR spectroscopy has been reported for an intermediate presumed to be [Ru(κ^2^-bpy)_2_(κ^1^-3,3′-dmbpy)(NCMe)]^2+^ (3,3′-dmbpy=3,3′-dimethyl-2,2′-bipyridyl).[6] Recent theoretical calculations have suggested that the initial ^3^MC state in [Ru(bpy)_3_]^2+^ type complexes in fact involves the elongation of two mutually *trans* Ru–N bonds and the formation of the four coordinate species [Ru(κ^2^-bpy)(κ^1^-bpy)_2_]^2+^.[7] It was proposed that it is this intermediate that is trapped by solvent and may then undergo ligand loss reactions.

We have previously reported the synthesis, characterization and photophysical investigation of the complexes [Ru(bpy)_3−*n*_(btz)_*n*_][PF_6_]_2_ (*n*=1–3).[8] The UV/Vis absorption spectra of [Ru(bpy)(btz)_2_]^2+^ and [Ru(bpy)_2_(btz)]^2+^ exhibited clearly defined ^1^MLCT bands, which are blueshifted relative to that of [Ru(bpy)_3_]^2+^ indicating MLCT state destabilization. DFT data suggested that the T_1_ states of [Ru(bpy)(btz)_2_]^2+^ and [Ru(bpy)_2_(btz)]^2+^ are of bpy-centred ^3^MLCT in character; however, that of [Ru(btz)_3_]^2+^ was found to be ^3^MC in character. Indeed, in agreement with the results of Alary et al.,[7] the optimized geometry for the T_1_ state of [Ru(btz)_3_]^2+^ was found to have a four coordinate [Ru(κ^2^-btz)(κ^1^-btz)_2_]^2+^ structure.

Therefore, we suspected that the destabilisation of the ^3^MLCT state in the heteroleptic complexes might promote photochemical reactivity through increased efficiency of ^3^MC state population. Indeed, we had noted in this earlier report that NMR samples of [Ru(bpy)(btz)_2_]^2+^ in [D_3_]acetonitrile that had been left to stand on the bench under ambient illumination revealed evidence of such photochemical conversion having occurred. Subsequent investigations revealed that the complex undergoes photoinitiated loss of one of the btz ligands in the absence of any steric promotion to yield the bis(solvento) complex *trans*-[Ru(bpy)(btz)(NCMe)_2_]^2+^. Further, and far more significantly, this proceeds via the unique photoreactive intermediate complex *trans*-[Ru(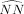)(κ^2^-btz)(κ^1^-btz)(NCMe)]^2+^, which forms quantitatively from the starting complex in a matter of minutes and exhibits a lifetime of several hours. A preliminary communication of this work has been recently published.[9]

Herein, we report these subsequent results detailing the photoreactivity of analogous bis(bitriazolyl) diimine complexes. Further, we present the crystallographic characterization of the intermediate complex *trans*-[Ru(bpy)(κ^2^-btz)(κ^1^-btz)(NCMe)][PF_6_]_2_.

## Results and Discussion

**Scheme 1:**
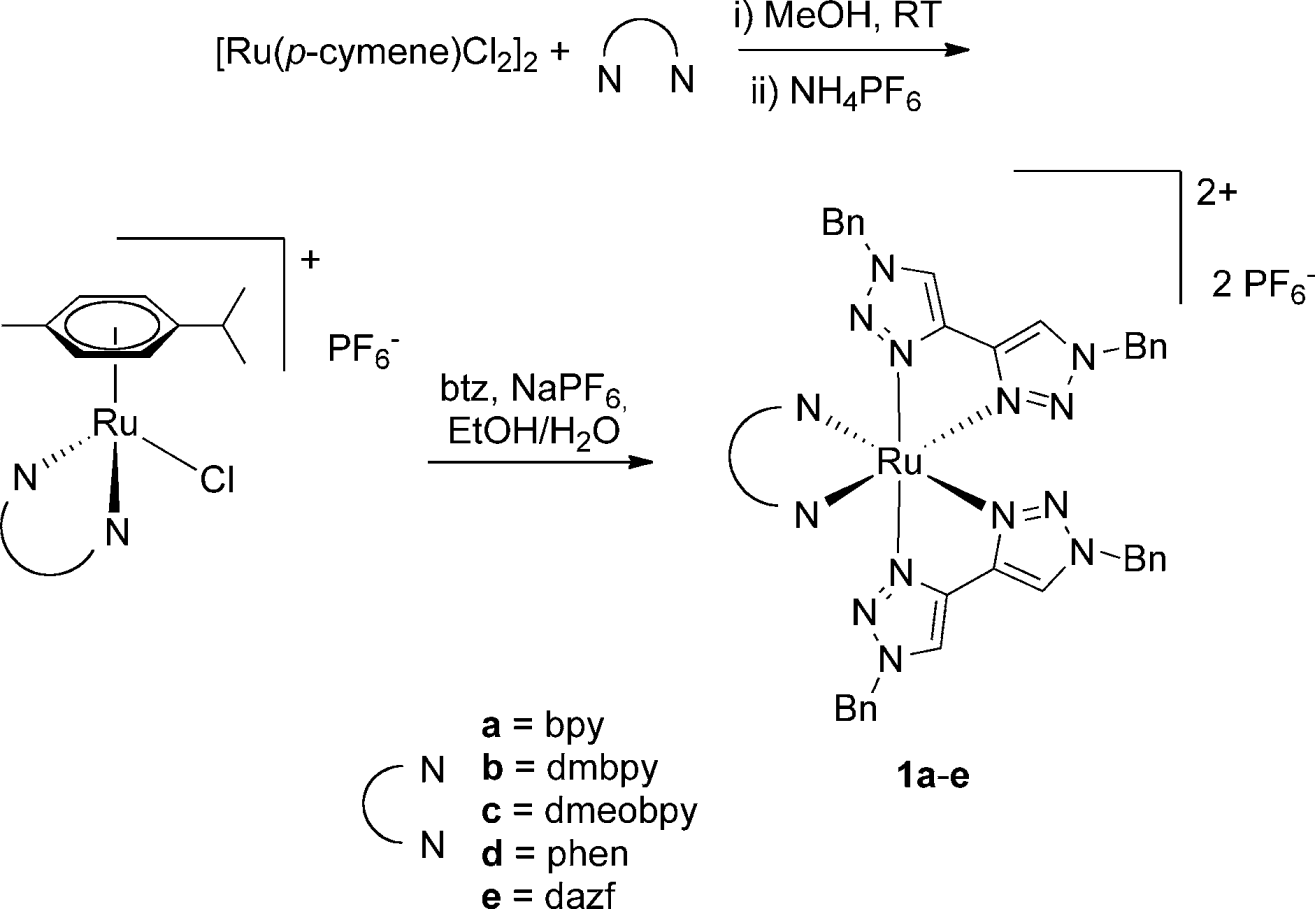
Synthesis of complexes [Ru(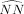)(btz)_2_][PF_6_]_2_ (1 a–e).

**Scheme 2:**
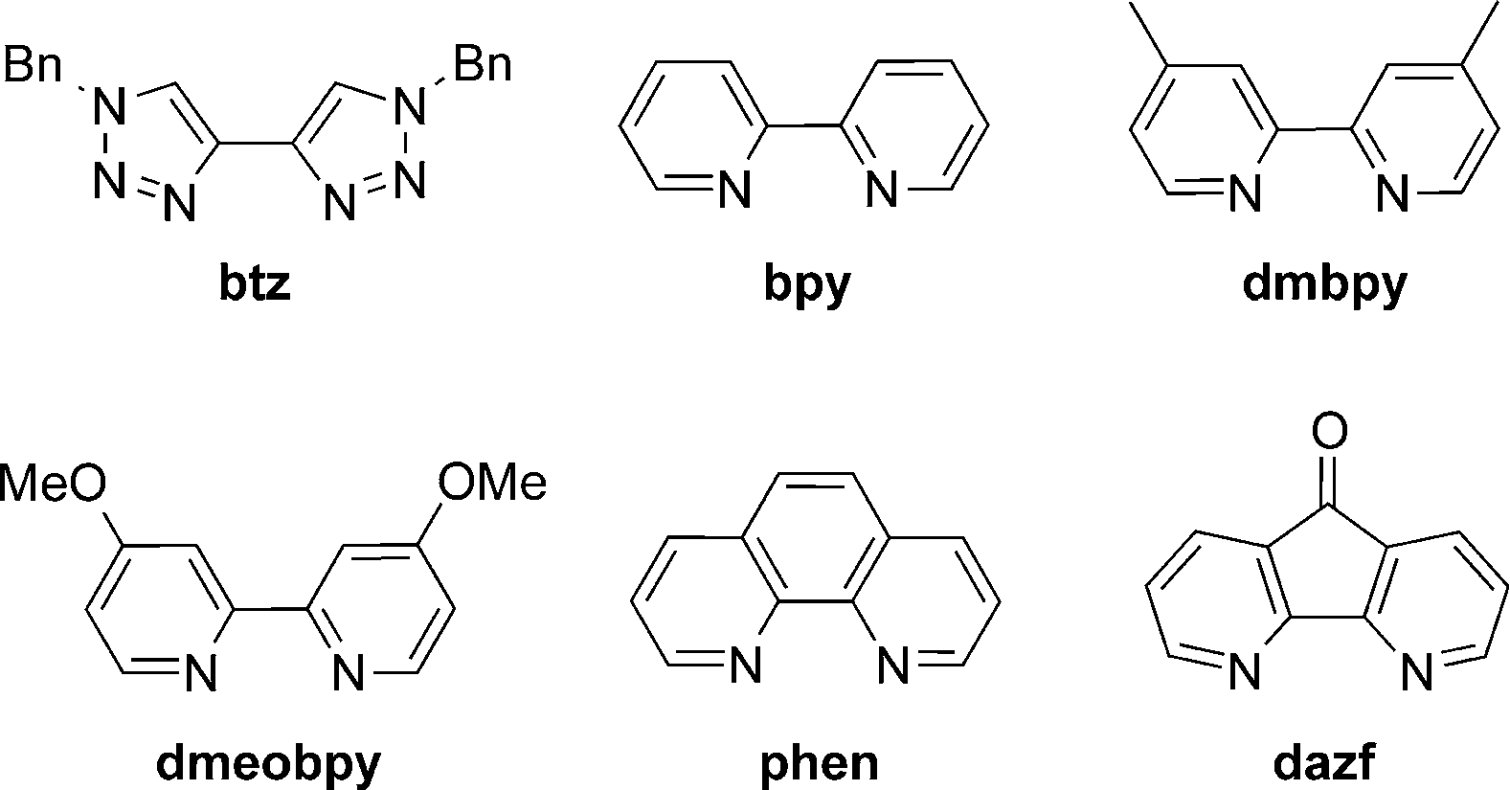
Structures and naming of ligands.

The complexes [Ru(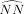)(btz)_2_][PF_6_]_2_ (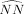
=dmbpy (**1 b**), dmeobpy (**1 c**), phen (**1 d**), dazf (**1 e**); see Schemes 1 and 2) were prepared by an analogous route to that for [Ru(bpy)(btz)_2_][PF_6_]_2_ (**1 a**) already reported.[8] Briefly, the diimine ligand 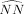 is coordinated to the metal by reaction with [Ru(*p*-cymene)(Cl)_2_]_2_. The desired product complexes were then prepared by reaction of the resultant half-sandwich complexes with two equivalents of btz upon heating at reflux in ethanol/water in the presence of sodium hexafluorophosphate (Scheme 1).

In each case, the ^1^H NMR spectra exhibit signals, which indicate that the two donor rings of the 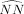 ligand, are magnetically equivalent. Additionally, two singlet resonances were also observed corresponding to the triazole ring protons of the btz ligands reflecting the *C*_2_ symmetry of the complexes.

**Figure 1:**
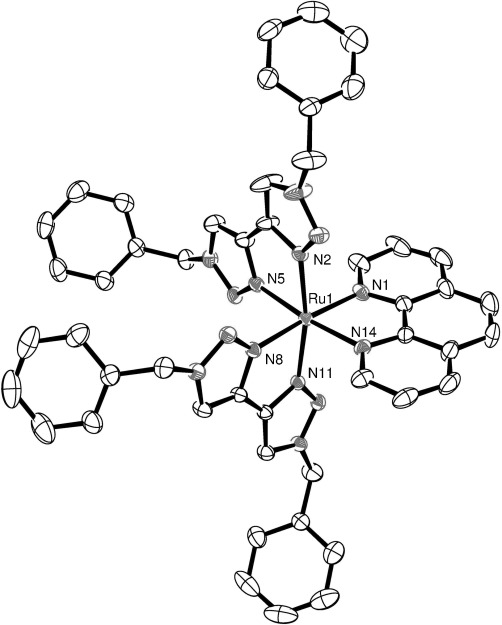
ORTEP plot of the structure of the cation [Ru(phen)(btz)_2_]^2+^ (1 d, hydrogen atoms, counterions and solvent molecules were removed for clarity, ellipsoids are drawn at 50 % probability level).

**Figure 2:**
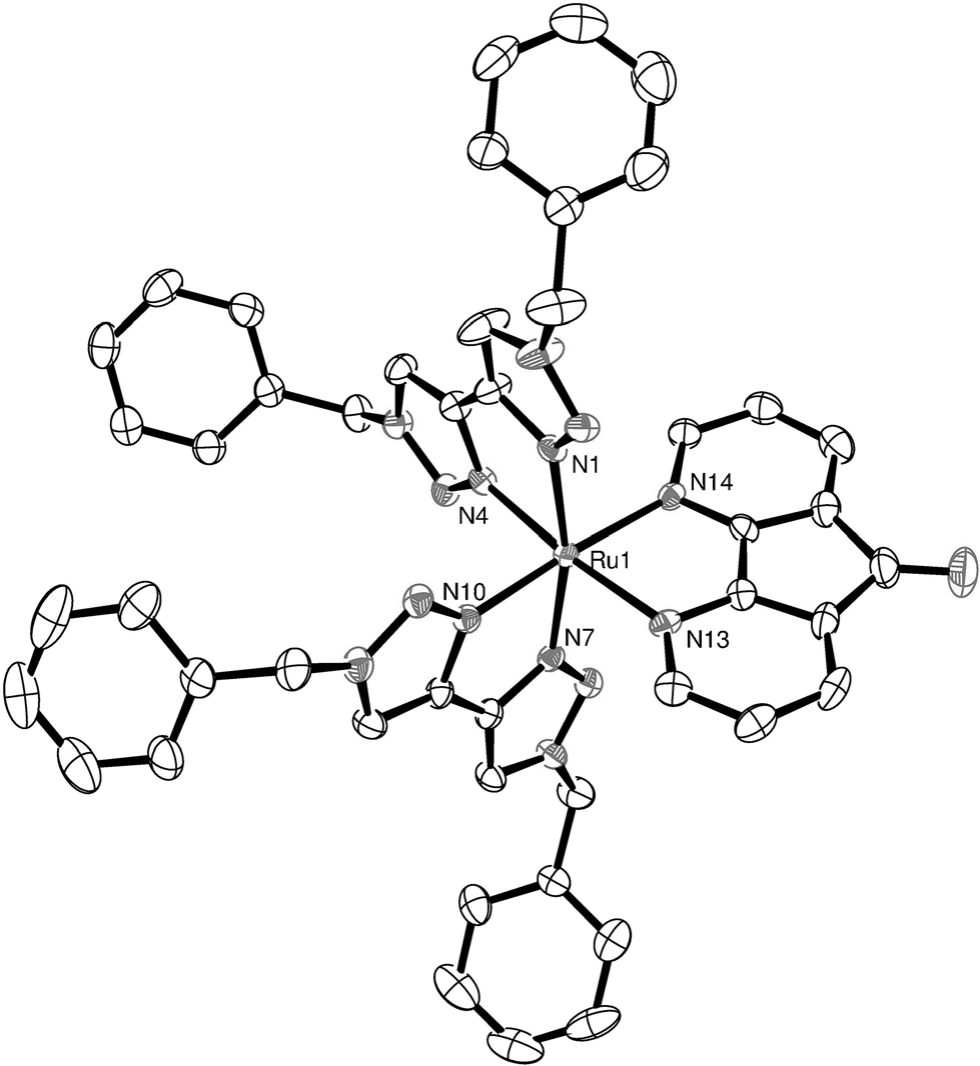
ORTEP plot of the structure of the cation [Ru(dazf)(btz)_2_]^2+^ (1 e, hydrogen atoms, counterions and solvent molecules were removed for clarity, ellipsoids are drawn at 50 % probability level).

Crystals of diffraction quality for **1 d** and **e** were grown by slow vapour diffusion of diethyl ether into acetonitrile solutions of the complexes in the dark. Molecular structures of the cations are shown in Figures 1 and 2, respectively, and selected bond lengths and angles are presented in Table 1. Both complexes crystallize in the space group *P42bc* with a solvent molecule incorporated. In the case of **1 e**, this is a molecule of acetonitrile; however for **1 d**, there is coincident and disordered acetonitrile and diethyl ether, which was successfully modelled with partial occupancy in a ratio of 47:53.

**Table 1:** Selected bond lengths [Å] and angles [°] for crystal structures for complexes 1 d, e, 3 a and d

[Ru(phen)(btz)_2_][PF_6_]_2_ (1 d)				*trans*-[Ru(bpy)(btz)(MeCN)_2_][PF_6_]_2_ (3 a)			
Ru–N(1)	2.061(2)	N(1)-Ru-N(14)	79.88(8)	Ru–N(1)	2.051(1)	N(1)-Ru-N(2)	79.04(6)
Ru–N(2)	2.063(2)	N(2)-Ru-N(5)	77.79(8)	Ru–N(2)	2.053(1)	N(3)-Ru-N(6)	76.67(5)
Ru–N(5)	2.057(2)	N(8)-Ru-N(11)	77.78(8)	Ru–N(3)	2.078(1)	N(1)-Ru-N(6)	177.38(6)
Ru–N(8)	2.050(2)	N(1)-Ru-N(8)	175.37(9)	Ru–N(6)	2.097(1)	N(2)-Ru-N(3)	179.51(6)
Ru–N(11)	2.064(2)	N(2)-Ru-N(11)	164.84(8)	Ru–N(9)	2.018(2)	N(9)-Ru-N(10)	178.45(6)
Ru–N(14)	2.064(2)	N(5)-Ru-N(14)	173.19(9)	Ru–N(10)	2.021(2)		

[Ru(dazf)(btz)_2_][PF_6_]_2_ (1 e)				*trans*-[Ru(phen)(btz)(MeCN)_2_][PF_6_]_2_ (3 d)			
Ru–N(1)	2.069(4)	N(1)-Ru-N(4)	79.9(1)	Ru–N(1)	2.058(2)	N(1)-Ru-N(2)	80.18(6)
Ru–N(4)	2.031(4)	N(7)-Ru-N(10)	77.9(1)	Ru–N(2)	2.061(2)	N(3)-Ru-N(6)	77.11(6)
Ru–N(7)	2.069(4)	N(13)-Ru-N(14)	82.2(2)	Ru–N(3)	2.066(2)	N(1)-Ru-N(6)	177.04(6)
Ru–N(10)	2.035(4)	N(1)-Ru-N(7)	167.4(1)	Ru–N(6)	2.093(2)	N(2)-Ru-N(3)	179.59(6)
Ru–N(13)	2.128(4)	N(4)-Ru-N(13)	174.2(2)	Ru–N(9)	2.027(2)	N(9)-Ru-N(10)	176.63(7)
Ru–N(14)	2.112(4)	N(10)-Ru-N(14)	176.2(2)	Ru–N(10)	2.024(2)		

Both complexes exhibit a distorted octahedral geometry resulting from the constraint inherent in the chelating ligands. The Ru–N distances in **1 d** are all typical for this type of complex with an average bond length of 2.06 Å. For comparison, the average Ru–N distance for [Ru(btz)_3_]Cl_2_ reported by Monkowius[10] is 2.05(3) Å, and for [Ru(bpy)_3_]^2+^ it is 2.056(2) Å.[11] The two Ru–N distances for the dazf of **1 e** ligand are longer (2.128(4) and 2.112(4) Å) and are similar in structure to that observed in the crystal structure of [Ru(bpy)_2_(dazf)][ClO_4_]_2_, reported by Yang et al.[12]

**Figure 3:**
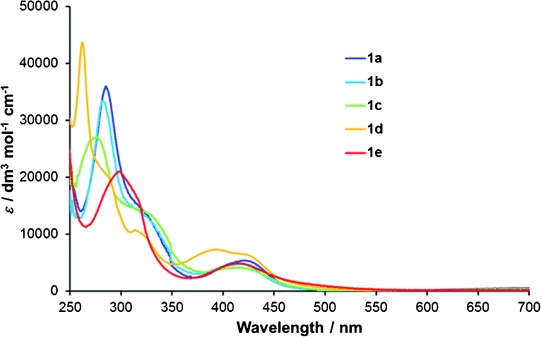
UV/Vis absorption spectra for complexes 1 a–e in acetonitrile (1.7×10^−5^ mol dm^−3^).

UV/Vis absorption spectra were recorded for complexes **1 a**–**e** in acetonitrile solutions and are presented in Figure 3. Wavelengths of major bands are summarized in Table 2. All complexes exhibited intense bands assigned to diimine ligand-centred π→π* transitions below *λ*=270 nm with that of **1 e** appearing below 250 nm. Weaker ^1^MLCT bands were observed between 350 and 500 nm. Similar to the spectrum for **1 a**, the spectra of **1 b** and **d** contain shoulders on the π→π* band assigned to ^1^MLCT transitions with charge transfer to the btz ligands. Complex **1 e** exhibits a broad band in this region centred at around 300 nm.

**Table 2:** Summarised UV/Vis absorption data for complexes 1 a–e in acetonitrile solutions

Complex	*λ* [nm] (*ε* [dm^3^ mol^−1^ cm^−1^])
**1 a**	286 (36000) and 425 (5360)
**1 b**	282 (33400), 324 (13100) and 419 (4720)
**1 c**	276 (27100), 323 (14000) and 418 (4080)
**1 d**	262 (43600), 286 (20400), 397 (7240) and 428 (6040)
**1 e**	302 (20300) and 419 (4810)

The MLCT bands of **1 b** and **c** are blueshifted relative to that of **1 a** consistent with the destabilization of the bipyridyl-centred LUMO with incorporation of the electron-donating methyl and methoxy substituents, respectively. The MLCT bands for **1 d** and **e** appear in a similar region to that of **1 a** though that of **1 d** is much broader with maxima at *λ*=397 and 428 nm. The MLCT band of **1 e** appears slightly blueshifted relative to the lower-energy maximum of the band for **1 d**; however the band has a more significant absorption tailing beyond 500 nm. This would be consistent with the stabilization of the LUMO and hence the S_1_^1^MLCT state due to the electron-withdrawing carbonyl moiety of the dazf ligand.

**Figure 4:**
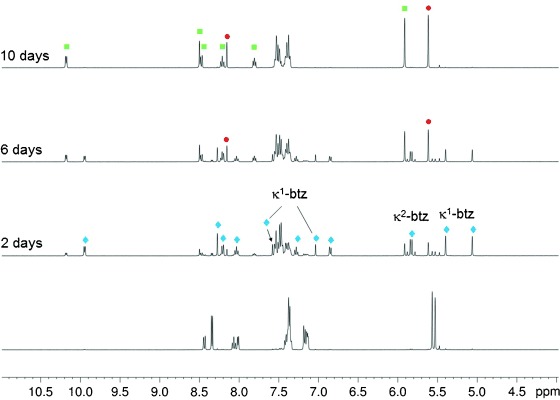
^1^H NMR spectra recorded during the photolysis of a sample of 1 a in [D_3_]acetonitrile left in ambient daylight (•=free btz, ⧫=2 a, ▪=3 a).

When left to stand in ambient light, NMR samples (typically, 8.5 mmol dm^−3^) of **1 a** in [D_3_]acetonitrile revealed new resonances indicative of conversion into new complexes. Representative ^1^H NMR spectra for the conversion of a fresh sample of **1 a** in [D_3_]acetonitrile, deliberately left exposed to incident daylight over ten days, are presented in Figure 4.

**Scheme 3:**
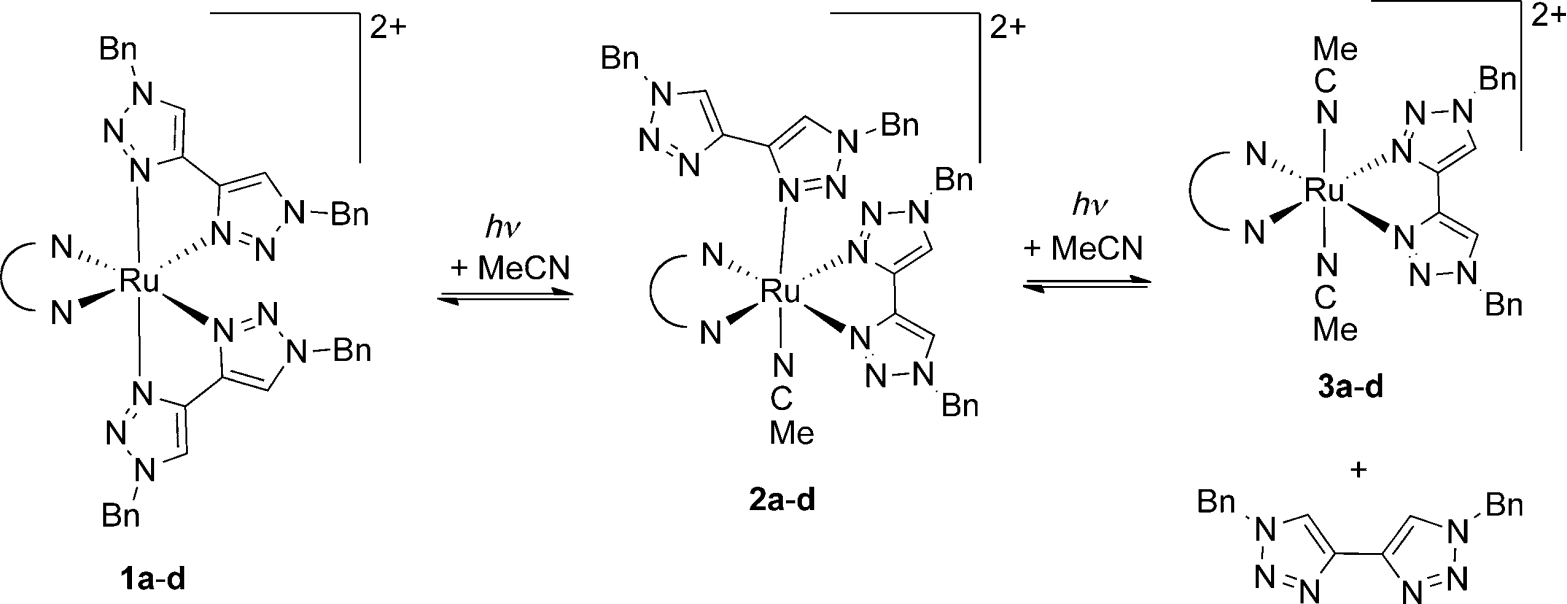
Photochemical conversion of complexes 1 a–d to 3 a–d via intermediates 2 a–d.

Examination of these spectra indicate that the samples undergo clean conversion of **1 a** into a new complex **3 a** and that this conversion proceeds via an intermediate species **2 a** (Scheme 3). The spectroscopic elucidation of the structures of these product and intermediate complexes is discussed below. Samples of **1 b**–**d** were observed to undergo similar conversions to give analogous products **3 b**–**d** via analogous intermediate species **2 b**–**d**. Samples left in the dark at room temperature and also in the dark in a refrigerator did not undergo this conversion, demonstrating that this is a photochemical rather than thermally driven process.

It was found that these photochemical conversion processes could be greatly accelerated by suspending the NMR sample between the fluorescent tubes of a domestic 23 W 1450 lumen light bulb and utilizing an electric fan to maintain sample temperatures at approximately 40 °C. This simple experimental set-up allowed these photochemical conversion processes to be monitored with the sample periodically removed for NMR interrogation before returning to the photolysis set-up. Under these conditions, complete conversion of complexes **1 a**–**d** to the intermediate species **2 a**–**d** was observed to occur in 3–6 min, whilst full conversion to **3 a**–**d** and ejection of free btz occurs over the time of one to two days.

The ^1^H NMR spectrum of the photochemical product complex **3 a** in [D_3_]acetonitrile showed four resonances for the bpy ligand demonstrating that the two pyridine rings maintain magnetic equivalence. In addition to the signals for the free btz ligand, signals were also observed for a second btz ligand, still coordinated to the metal. A singlet resonance was observed at *δ*=8.50 ppm with a relative integration corresponding to two protons for the hydrogen atoms of triazole rings. A further singlet for the benzyl substituent methylene protons was observed at *δ*=5.91 ppm. These data are indicative of the magnetic equivalence of the two triazole rings of the btz ligand. This, combined with the symmetry observed for the bpy ligand, mandates a co-planar arrangement of these two ligands with presumably two mutually *trans* solvent molecules occupying the remaining coordination sites. Therefore, this complex is assigned as having the structure *trans*-[Ru(bpy)(btz)(NCMe)_2_]^2+^. Samples of **1 a** in CD_2_Cl_2_ did not show this conversion; however, resonances for **2 a** and **3 a** were observed upon addition of 50 μL of CD_3_CN and after subsequent illumination. Therefore, this corroborates the assumption that **2 a** and **3 a** are acetonitrile solvento complexes. For a sample of **1 a** that had fully converted to **3 a** in CH_3_CN examination by electrospray mass spectrometry showed the presence of dications with *m*/*z* 328.1, 307.6 and 287.1 corresponding to the species [Ru(bpy)(btz)(NCMe)_2_]^2+^, [Ru(bpy)(btz)(NCMe)]^2+^ and [Ru(bpy)(btz)]^2+^, respectively. Additionally, the monocationic ion pair {[Ru(bpy)(btz)(NCMe)_2_][PF_6_]}^+^ was also observed (*m*/*z* 801.1). Each of these signals showed the expected isotope pattern for a mononuclear ruthenium complex.

**Figure 5:**
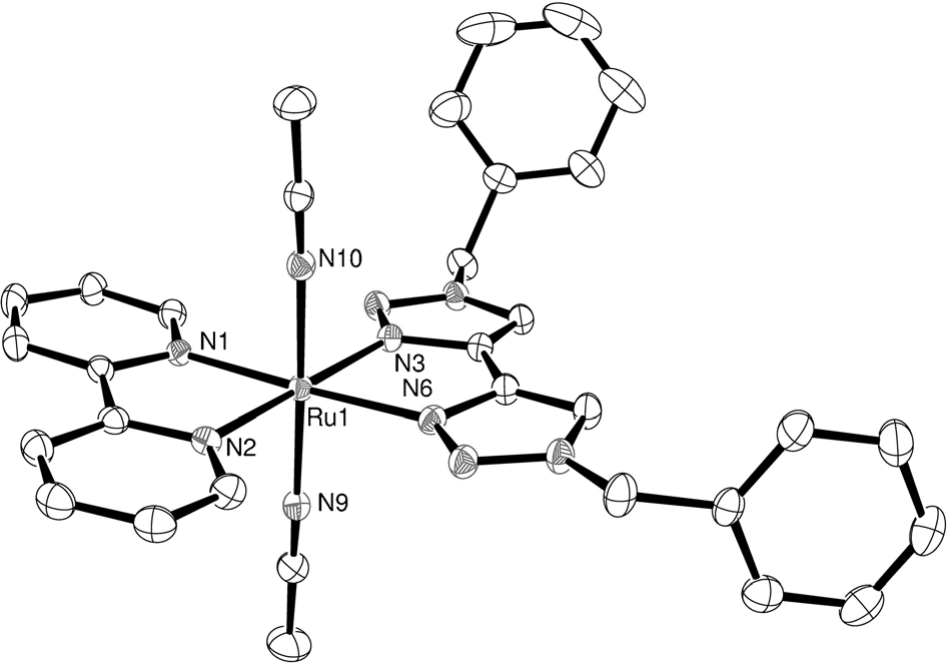
ORTEP plot of the structure of the cation [Ru(bpy)(btz)(MeCN)_2_]^2+^ (3 a, hydrogen atoms and counterions were removed for clarity, ellipsoids are drawn at 50 % probability level).[9]

**Figure 6:**
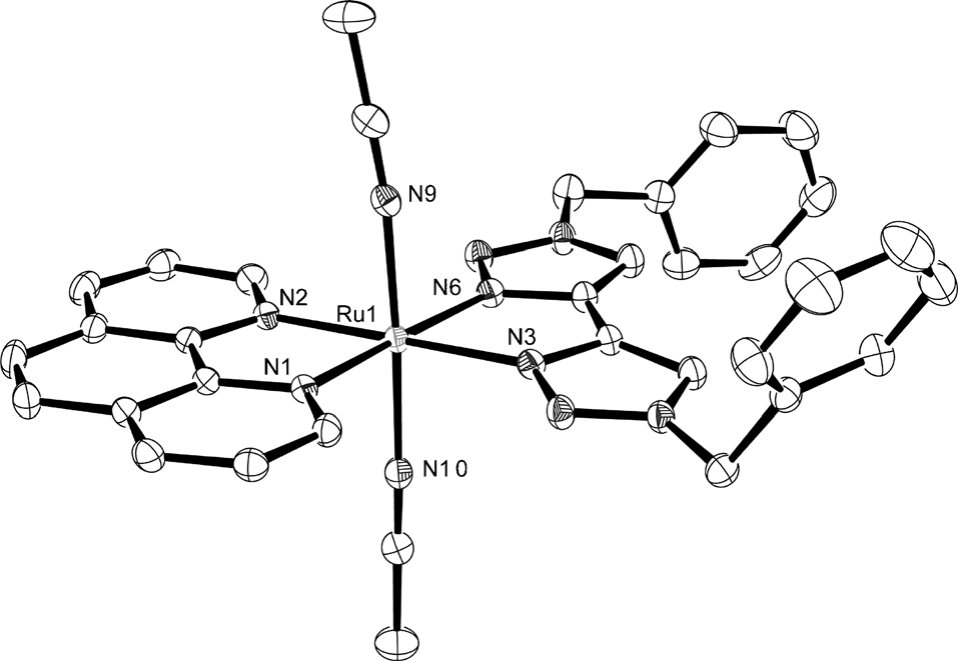
ORTEP plot of the structure of the cation [Ru(phen)(btz)(MeCN)_2_]^2+^ (3 b, hydrogen atoms and counterions were removed for clarity, ellipsoids are drawn at 50 % probability level).

After prolonged illumination of concentrated samples of **1 a** and **d**, which had fully converted to **3 a** and **d**, respectively, free btz was observed to precipitate. The liquor was decanted from these samples, the solvent removed and redissolved in CH_3_CN. These solutions were left in ambient daylight with slow vapour diffusion of diisopropyl ether for **3 a** and diethyl ether for **3 d**, which resulted in the formation of crystals suitable for X-ray diffraction analysis. Crystallographic data for **3 a** have been reported previously.[9] Structures of the cations for these complexes are shown in Figures 5 and 6. Selected bond lengths and angles are provided in Table 1. Growth of crystals for complexes **3 b** and **c** was also attempted, but efforts proved to be unsuccessful.

Compound **3 a** was found to crystallize in the space group *P*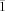, whereas **3 d** crystallized in the space group *P*21/*c*. Both exhibited distorted octahedral geometries and adopt Ru–N bond lengths and angles that are within normal limits for these ligands. The Ru–N distances of the bpy or phen ligands are shorter than those of the btz ligands at a range of 2.05–2.06 Å compared to 2.07–2.10 Å, respectively. The Ru–N(bpy) distances of **3 a** (2.051(1) and 2.053(1) Å) are slightly shorter than those of *trans*-[Ru(bpy)_2_(NCMe)_2_][ClO_4_]_2_ (Ru–N(bpy) 2.070(3), 2.068(3) Å),[13] yet the Ru–N(NCMe) distance is slightly longer (2.018(2) and 2.021(2) Å compared to 2.008(4) Å). These structures did not exhibit any bowed distortions of the chelate ligands, which is a notable feature of the *trans*-[Ru(bpy)_2_(X)_2_]^2+^ complexes (X=MeCN, NH_3_) and *trans-*[Ru(phen)_2_(py)_2_]^2+^ that results from steric crowding of the α-hydrogen atoms of the two diimine ligands.[13–14]

It is presumably the lack of any α-hydrogen substituents of the btz ligand that therefore facilitates the observed ligands rearrangement toward coplanarity. DFT calculations (see the Supporting Information) were carried out on both *cis* and *trans* isomers of **3 a** and [Ru(bpy)_2_(NCMe)]^2+^. These gas-phase calculations reproduced the distortions in *trans*-[Ru(bpy)_2_(NCMe)]^2+^ that were observed crystallographically and showed that it is approximately 40 kJ mol^−1^ less stable than its *cis* isomer. In contrast, *trans*-**3 a** was calculated to be 7.76 kJ mol^−1^ more stable than *cis*-**3 a**, thus accounting for the observed stereochemical preference.

Curiously, complex **1 e** did not undergo any observable photochemical conversion even when heated to 80 °C during illumination. As we have stated earlier, we reasoned that elevation of the ^3^MLCT state in these btz complexes might increase their propensity to undergo photochemical reaction through greater efficiency of ^3^MC state population. Indeed, the ready photolability of **1 a–d** would appear to confirm this. The lack of comparable reactivity for **1 e** could be a consequence of the stabilization of the dazf-centred LUMO and hence ^3^MLCT state compared to those of the other complexes, thus making the ^3^MC state inaccessible to thermal population. Alternatively, one might speculatively suggest that the presence of the strong C=O oscillator in the dazf ligand may result in efficient ^3^MLCT state quenching through vibrational relaxation pathways.

Structural identification of the intermediate complexes **2 a**–**d** became trivial after stereochemical characterization of **3 a**–**d** had been achieved. The intermediate species **2 a**–**d** exhibited similar resonances for the diimine ligands to those of **3 a**–**d**. Each complex also exhibited a sole singlet resonance for the triazole ring protons of a bidentate btz ligand between *δ*=8.25 and 8.35 ppm indicating the same co-planar arrangement as found for the final ligand-ejection-product complexes. However, the methylene protons of these bidentate btz ligands in **2 a**–**d** gave rise in each case to a geminal pair of doublets with significant roofing centred at approximately *δ*≈5.8 ppm (*J*_HH_ 15 Hz in all cases). Therefore, this indicates that the two ligands mutually *trans* to one another, above and below this plane, are different. In each case, further resonances were observed for a second coordinated btz ligand; two singlet resonances were observed, each with a relative integration corresponding to a single proton each, for the triazole ring protons in the ranges *δ*=6.88–7.13 and 7.58–7.60 ppm (the more de-shielded of these two resonances for the phenanthroline complex **2 d** is obscured by the multiplet arising from the phenyl protons of the benzyl substituents). In addition, a further two singlet resonances for the methylene protons of the second btz ligand in **2 a**–**d** and appear at *δ*=4.99–5.09 ppm and 5.31–5.43 ppm.

The magnetic inequivalence of the two triazole moieties in this second btz ligand and the co-planar arrangement of the diimine and the bidentate btz ligand demand that it should be coordinated in a monodentate fashion through one triazole ring. Therefore, we assign the intermediates **2 a–d** as having the unique structure *trans*-[Ru(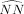)(κ^2^-btz)(κ^1^-btz)(NCMe)]^2+^. Electrospray mass spectrometry of partially converted samples of **1 a** in CH_3_CN showed dications with *m*/*z* of 445.1 and 465.6 corresponding to the cation of the starting material and the intermediate [Ru(bpy)(κ^2^-btz)(κ^1^-btz)(NCMe)]^2+^, respectively.

**Figure 7:**
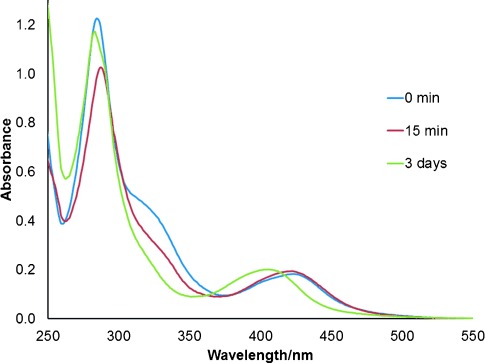
UV/Vis absorption spectra recorded during photolysis of 1 a in acetonitrile. At 15 min and three days, the sample contains predominantly 2 a and 3 a, respectively, based on NMR analysis.

UV/Vis spectra recorded during the photolysis in acetonitrile showed a bleaching of the shoulder at about *λ*≈300 nm for **1 a** attributed to transitions involving btz-centred MLCT character within 15 min at which time NMR analysis revealed the dominant species to be **2 a** (Figure 7). This was accompanied by a slight blueshift in the MLCT band from *λ*=425 to 421 nm. After three days of irradiation, during which time the sample had entirely converted to **3 a**, the shoulder at 300 nm was observed to bleach further consistent with the formal loss of one of the btz ligands. The low-energy MLCT band of **3 a** was significantly blueshifted with respect to those of **1 a** and **2 a** and appeared at *λ*=406 nm. The large blueshift in the MLCT band on conversion of **2 a** to **3 a** likely resulted from the increased mixing of btz orbital character in, and destabilization of the excited state due to the co-planarity that will enable efficient π communication through the ruthenium centre. The comparably small blueshift in the MLCT band upon conversion of **1 a** to **2 a** possibly arises due to deviations from coplanarity of the κ^2^-bpy and κ^2^-btz ligands due to the steric bulk of the κ^1^-btz ligand (see below). This would serve to disrupt communication between the two bidentate ligands and result in a far less destabilized MLCT state. Similar changes were observed for the other complexes in the series.

The κ^1^-btz complexes **2 a**–**d** appeared to be remarkably stable in solution at room temperature, reverting back to starting material only very slowly when samples are left in the dark. The approximate rate of this process was investigated by taking the samples used to monitor the forward process (which had been irradiated for ca. 6 h) and leaving them in the NMR spectrometer with a spectrum recorded every 20 min at 40 °C for 6 to 7 h. The conversion of complexes **2 a–d** to **1 a–d** was found to follow first-order kinetics with *k*_obs_ for all complexes in the region of 1.4×10^−5^ s^−1^. Over the course of these reversal reactions, the re-coordination of btz to complexes **3 a–d** that had formed during photolysis to reform **2 a**–**d** was found to be negligibly slow. Indeed, a fully converted foil-wrapped sample containing solely **3 a** and free btz was left in the dark for a fortnight and showed no noticeable change to the appearance of the ^1^H NMR spectrum. Therefore, it would appear that the overall conversion of **1 a–d** to **3 a–d** requires two photons; one to effect btz dechelation and form the intermediate, and the second to ultimately eject the κ^1^-btz ligand.

The resonances for the benzyl substituent phenyl-ring protons in complexes **1 a**–**d** and **3 a**–**d** have the appearance of overlapping, unresolved multiplets. However, cleanly resolved resonances were observed for the *ortho-* and *meta*-positions of one of the benzyl substituents of the κ^1^-btz ligand (for **2 a**­ *δ*=6.85 and 7.30, respectively). We had reasoned in our earlier communication[9] that this may indicate the formation of some intramolecular interaction, for example, by π stacking that possibly accounts for the stability of the intermediates **2 a**–**d** with respect to rapid reversion back to **1 a**–**d** or to final ligand ejection and formation of the products **3 a**–**d**. No evidence from NOESY spectroscopy could be found to corroborate this. In order to test this, complexes analogous to **1 a**, in which the btz ligand have less flexible phenyl substituents (**1 a^Ph^**) or propyl substituents (**1 a^Pr^**) that are incapable of π stacking, were prepared, and their photoreactive behaviour was monitored. For both complexes, identical behaviour was observed to those of the benzyl-substituted btz complexes with formation of stable κ^1^-btz intermediates. Although this would still account for the spectroscopic observations, we thus discounted our initial speculative suggestion that the aforementioned intramolecular interactions are responsible for the observed stability of the κ^1^-btz intermediate complexes. The primary reason for the stability of these intermediates would appear to stem from the rearrangement of the two ligands that remain bidentate, which inhibits re-chelation of the κ^1^-btz ligand. As a result, the κ^1^-btz ligand has no site *cis* to it occupied by a potentially labile solvento ligand.

**Figure 8:**
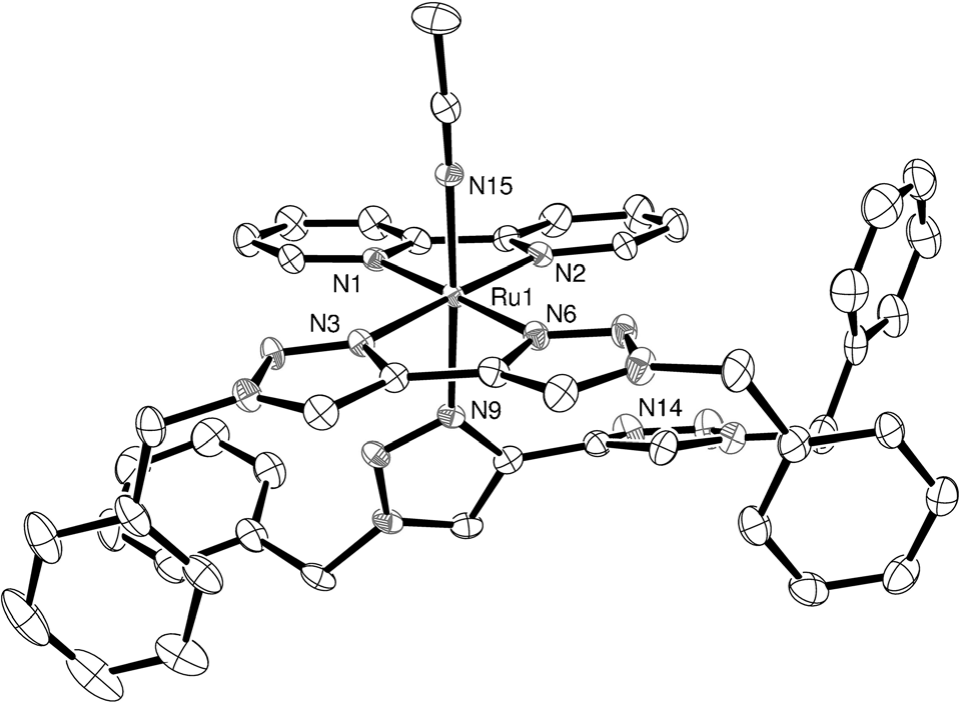
ORTEP plot of the structure of the cation *trans-*[Ru(bpy)(κ^2^-btz)(κ^1^-btz)(MeCN)]^2+^ (2 a, hydrogen atoms, solvent and counterions were removed for clarity, ellipsoids are drawn at 50 % probability level).

Because the intermediates **2 a–d** appeared to show remarkable stability, this raised the question of whether the κ^1^-btz intermediate can be isolated. Fresh, concentrated NMR samples of **1 a** were irradiated in the lamp for 15 min, after which their ^1^H NMR spectra were examined to confirm total conversion to the intermediate **2 a**. The samples were then decanted into small open vials, which in turn were placed within larger vials for vapour diffusion of diethyl ether to effect crystallization. Further, these samples were placed in the refrigerator to slow the rate of reversion of the intermediate back to the starting material. Several plate-like crystals suitable for X-ray diffraction analysis were obtained by this method. The molecular structure of the cation of **2 a** is presented in Figure 8 and comprehensively confirms the identity of the complex as the proposed κ^1^-btz ligand-loss intermediate.

The complex crystallizes in the *P*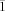 space group and, in addition to one coordinated acetonitrile, there are an additional 2.5 molecules of acetonitrile per cation in the unit cell. Similar to the structures of **3 a** and **d**, the bpy ligand and the κ^2^-btz ligand are arranged in an approximately co-planar fashion. In agreement with spectroscopic data, the complex contains one acetonitrile ligand *trans* to the κ^1^-btz ligand. The planes of the triazole rings of the monodentate btz ligand are twisted relative to each other with a N-C-C-N torsion angle of 64.9°. The Ru–N bond lengths to the bidentate ligands are similar to those of **3 a** and **d** (Table 3).

**Table 3:** Selected bond lengths [Å] and angles [°] for the X-ray crystal structure of *trans*-[Ru(bpy)(κ^2^-btz)(κ^1^-btz)(NCMe)][PF_6_]_2_⋅2.5 MeCN (2 a⋅2.5 MeCN)

Ru(1)–N(1)	2.063(3)	N(1)-Ru(1)-N(2)	78.64(12)
Ru(1) –N(2)	2.059(3)	N(3)-Ru(1)-N(6)	77.08(11)
Ru(1)–N(3)	2.093(3)	N(9)-Ru(1)-N(15)	177.11(11)
Ru(1)–N(6)	2.102(3)	N(1)-Ru(1)-N(6)	178.87(10)
Ru(1)–N(9)	2.081(3)	N(2)-Ru(1)-N(3)	178.55(11)
Ru(1)–N(15)	2.020(3)		

Evidence from UV/Vis absorption spectra for the existence of an unstable intermediate, assigned as [Ru(κ^2^-bpy)_2_(κ^1^-bpy)(NCS)]^+^, which forms during what appears to be a stepwise photolysis of [Ru(bpy)_3_][NSC]_2_ to [Ru(bpy)_2_(NCS)_2_], has also been reported.[5] A solvento intermediate corresponding to the formula [Ru(bpy)_2_(3,3′-dmbpy)(NCMe)]^2+^ has been detected at very low concentrations by electrospray mass spectrometry with additional ^1^H NMR evidence.[6] Herein, the 3,3′-dimethyl-2,2′-bipyridyl (3,3′-dmbpy) ligand was assigned as being κ^1^-coordinated, *cis* to the acetonitrile solvento ligand. The formation here is presumably facilitated by the steric repulsion between the methyl substituents with re-chelation inhibited for the very same reason. A stable iridium(III) complex containing a κ^1^-bpy ligand, [Ir(bpy)_2_(κ^1^-bpy)(OH)_2_]^3+^, was previously reported by Watts et al.[15] However, when subsequently structurally characterized by Wickramasinghe et al. the complex proved to be a “rollover” cyclometalated complex, in which one of the bpy ligands is a 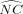 chelate with the non-coordinated N-atom protonated and hydrogen bonded to water.[16] To the best of our knowledge, **2 a** represents the only known example of a structurally characterized photochemical ligand-loss intermediate. The stability of the complex we ascribe to the concomitant new rearrangement of the bidentate bpy and btz ligand toward co-planarity. Enabled by the lesser steric demands of the btz over bpy, this then retards the re-chelation of the monodentate btz ligand.

Several triazole-containing complexes, which showed attractive photophysical properties, such as high-luminescent quantum yield, have been reported.[17] More recently, results from the group of Zysman-Colman have shown that the bis(cyclometalated) complex [Ir(dfptz)_2_(btz)]^+^ (dfptzH=1-benzyl-4-(2,4-difluorophenyl)-1,2,3-triazole) containing four triazole moieties undergoes photochemical btz ligand-loss reactions by a mechanism distinct from Ir–N(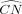) cleavage reactions known for similar complexes.[18] Although investigating the triazole-containing complexes [Ru(tap)_2_(pytz)]^2+^ and [Ru(tap)_2_(btz)]^2+^ (tap=tetraazaphenanthrene) as potential photooxidation agents, Mattiuzzi et al. demonstrated photochemical decomposition, presumably by ligand loss, by UV/Vis spectroscopy.[19] Although these and our results have important implications for the design of photoactive triazole-containing metal complexes, they also offer opportunities for fundamental investigation of the intriguing photophysics imparted by these ligands and their possible photolytic applications. Complexes of this type might find potential utility as photodynamic anti-cancer agents or as new photoactivated supramolecular synthons.

The results presented herein represent the first unequivocal spectroscopic characterization, and to the best of our knowledge, the first crystallographic characterization of a ligand-loss intermediate from a ruthenium tris(diimine)-type complex that contains a κ^1^-coordinated ligand. What is striking about this system is the ease with which the complex undergoes photochemical rearrangement to form the intermediate, occurring in the absence of any steric promoting groups to lower the ^3^MC state. Instead, the photolability appears to stem from the electronic tuning imparted by the btz ligand that destabilizes the ^3^MLCT state making the ^3^MC state easily accessible. Efforts are currently underway to carry out a detailed mechanistic study of the photochemical conversion process alongside a thorough theoretical characterization of the excited states involved. Results from these ongoing studies will be published in due course.

## Conclusion

We have reported here the unprecedented, unambiguous spectroscopic observation and structural characterization of a photoreactive ligand-loss intermediate solvento complex containing a monodentate chelate ligand from Ru^II^ tris(chelate) complexes of the form [Ru(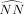)(btz)_2_]^2+^. These intermediate complexes form quantitatively from the starting materials in a matter of minutes and involve a new rearrangement such that the chelate ligands that remain bidentate become co-planar. This rearrangement engenders the intermediate complexes with remarkable stability to re-chelation of the monodentate btz ligand. As a consequence, this enabled the isolation of crystals of the intermediate complex *trans*-[Ru(bpy)(κ^2^-btz)(κ^1^-btz)(NCMe)][PF_6_]_2_ allowing the trapping and structural characterization of this complex.

The remarkably facile nature of the photoreactivity exhibited by these complexes, in which the 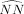 ligand is a phenanthroline or substituted bipyridine ligand, is ascribed to the btz-induced destabilization of the ^3^MLCT state such that ^3^MC population becomes efficient. In the case of the diazafluorenone analogue, in which no comparable photoreactivity was observed, stabilization of the ^3^MLCT state by the electron-withdrawing C=O group, and/or vibrational relaxation involving the same moiety resulting in rapid excited-state quenching thus inhibits ^3^MC state population.

Complexes based on those reported herein may have potential applications as light-activated anti-cancer drugs, photoinitiated supramolecular synthons for the construction of light-harvesting architectures and as components in light-activated molecular machines. Efforts to pursue these possibilities are currently underway, and results will be reported in due course.

## Experimental Section

Compound **1 a** and the ligand 1,1′-dibenzyl-4,4′-bi-1,2,3-triazolyl were prepared as previously described.[8] Precursors [Ru(*p*-cymene)(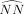)Cl][PF_6_] (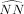
=dmbpy, phen; synthetic procedures are given in the Supporting Information for reference),[20] and the ligands diazafluorenone[21] and 1,1′-diphenyl-4,4′-bi-1,2,3-triazolyl[22] were prepared by literature methods. All other ligands were purchased from Aldrich Chemicals or Acros Organics and used as supplied.

UV/Vis absorption spectra were recorded on Varian Cary 300 or Agilent Cary 60 spectrometers. Samples were prepared in dimmed-light conditions and analysed immediately. NMR data were collected on Bruker 500 Avance and Bruker 400 Ascend spectrometers. Mass spectrometry was carried out on a Bruker Micro-Q-TOF instrument.

### Synthesis of [RuCl(*p*-cymene)(dmeobpy)][PF_6_]

[RuCl_2_(*p*-cymene)]_2_ (103.5 mg, 0.17 mmol) and 4,4′-dimethoxy-2,2′-bipyridyl (143.2 mg, 0.66 mmol, 3.9 equiv) were suspended in MeOH (10 mL), and the reaction mixture was vigorously stirred at RT for 3 h. After this time, a concentrated aqueous solution of NH_4_PF_6_ was added, and product precipitated as an orange-yellow solid. This was filtered and washed with Et_2_O (10 mL). The product was purified by recrystallisation from MeCN/Et_2_O. Yield 179.2 mg (84 %).

^1^H NMR (400 MHz, CD_3_CN): *δ*=1.03 (s, 3 H, CH(C*H*_3_)), 1.05 (s, 3 H, CH(C*H*_3_)), 2.22 (s, 3 H, *p*-cymene C*H*_3_), 2.64 (sp, ^3^*J*_HH_=7.0 Hz, 1 H, C*H*(CH_3_)_2_), 4.06 (s, 6 H, 4-O*Me*bpy), 5.65 (d, ^3^*J*_HH_=6.3 Hz, 2 H, *p*-cymene Ar-C*H*), 5.86 (d, ^3^*J*_HH_=6.3 Hz, 2 H, *p*-cymene Ar-C*H*), 7.24 (dd, ^4^*J*_HH_=2.8 Hz, ^3^*J*_HH_=6.6 Hz, 2 H, 4-OMebpy-*H*_5_), 7.81 (d, ^4^*J*_HH_=2.8 Hz 2 H, 4-OMebpy-*H*_3_), 9.07 (d, ^3^*J*_HH_=6.6 Hz, 2 H, 4-OMebpy-*H*_6_); ^13^C NMR (100.6 MHz, CD_3_CN): *δ*=18.5 (*C*H_3_, *p*-cymene), 21.8 (*C*H_3_, CH(*C*H_3_)_2_), 31.3 (CH, *C*H(CH_3_)_2_), 57.4 (*C*H_3_, 4-O*Me*bpy), 83.9 (*C*H, *p*-cymene Ar), 86.4 (*C*H, *p*-cymene Ar), 103.5 (*C*, *p*-cymene Ar-*C*(CH_3_)), 104.4 (*C*, *p*-cymene Ar-*C*CH(CH_3_)_2_), 110.7 (*C*H, 4-OMebpy-*C*_3_), 114.3 (*C*H, 4-OMebpy-*C*_5_), 156.5 (*C*, 4-OMebpy-*C*_6_), 156.6 (*C*, 4-OMebpy-*C*_2_), 168.8 (*C*H, 4-OMebpy-*C*_4_); HRMS (ESI): *m*/*z* calcd for [RuClO_2_N_2_C_22_H_26_]^+^ (486.97 g mol^−1^): 487.072082, found 487.073476.

### Synthesis of [RuCl(*p*-cymene)(dazf)][PF_6_]

[RuCl_2_(*p*-cymene)]_2_ (74.9 mg, 0.12 mmol) and 1,10-diazafluorenone (89.2 mg, 0.49 mmol, 4 equiv) were suspended in MeOH (7 mL), and the reaction mixture was vigorously stirred at RT for 3 h. After this time, an excess of NH_4_PF_6_ was added, and the volume of the solution was reduced by half in vacuo. An orange precipitate was formed; it was filtered and washed with Et_2_O (10 mL). The product was purified by recrystallisation from MeCN/Et_2_O (2:1). Yield 89.5 mg (61 %).

^1^H NMR (500 MHz, CD_3_CN): *δ*=1.21 (s, 3 H, CH(C*H*_3_)), 1.23 (s, 3 H, CH(C*H*_3_)), 2.21 (s, 3 H, *p*-cymene C*H*_3_), 2.86 (sp, ^3^*J*_HH_=6.9 Hz, 1 H, C*H*(CH_3_)_2_), 5.87 (d, ^3^*J*_HH_=6.3 Hz, 2 H, *p*-cymene Ar-C*H*), 6.05 (d, ^3^*J*_HH_=6.2 Hz, 2 H, *p*-cymene Ar-C*H*), 7.80 (dd, ^3^*J*_HH_=5.7 Hz, 7.6 Hz, 2 H, azf-*H*_5_), 8.25 (d, ^3^*J*_HH_=7.6 Hz, 2 H, azf-*H*_4_), 9.11 ppm (d, ^3^*J*_HH_=5.6 Hz, 2 H, azf-*H*_6_); ^13^C NMR (125.8 MHz, CD_3_CN): *δ*=18.5 (*C*H_3_, *p*-cymene), 21.8 (*C*H_3_, CH(*C*H_3_)_2_), 31.6 (CH, *C*H(CH_3_)_2_), 82.3 (*C*H, *p*-cymene Ar), 83.2 (*C*H, *p*-cymene Ar), 101.3 (*C*, *p*-cymene Ar-*C*(CH_3_)), 105.9 (*C*, *p*-cymene Ar-*C*CH(CH_3_)_2_), 130.2 (*C*H, azf-*C*_5_), 130.5 (*C*H, azf-*C*_3_), 135.6 (*C*H, azf-*C*_4_), 156.7 (*C*H, azf-*C*_6_), 165.5 (*C*, phen-*C*_2_), 185.8 ppm (*C*O); HRMS (ESI): *m*/*z* calcd for [RuOClN_2_C_21_H_20_]^+^ (452.91 g mol^−1^): 453.030217; found 453.030792.

### Synthesis of [Ru(dmbpy)(btz)_2_][PF_6_]_2_ (1 b)

[RuCl(*p*-cymene)(dmbpy)][PF_6_] (51.2 mg, 0.09 mmol) and btz (52.0 mg, 0.16 mmol, 1.9 equiv) and NaPF_6_ (60.0 mg, 0.36 mmol, 4.1 equiv) were added to a degassed solution of EtOH/H_2_O (8 mL; 3:1). The bright yellow reaction mixture was heated at 90 °C under N_2_ for 16 h. After cooling, a bright orange precipitate was formed. This was filtered and washed with Et_2_O (10 mL) and dried. Yield 51.6 mg (52 %).

^1^H NMR (400 MHz, CD_3_CN): *δ*=2.57 (s, 6 H, 4-*Me*bpy), 5.53 (s, 4 H, CH*H* of Bz), 5.56 (s, 4 H, CH*H* of Bz), 7.13–7.18 (m, 8 H, *Ph*), 7.23 (d, ^3^*J*_HH_=5.8 Hz, 2 H, 4-Mebpy*-H*_5_), 7.34–7.41 (m, 12 H, *Ph*), 7.79 (d, ^3^*J*_HH_=5.8 Hz, 2 H, 4-Mebpy-*H*_6_), 8.28 (s 2 H, 4-Mebpy-*H*_3_),8.33 (s, 2 H, C*H*N_3_), 8.34 ppm (s, 2 H, C*H*N_3_); ^13^C NMR (100.6 MHz, CD_3_CN): *δ*=20.8 (*C*H_3_, 4-*Me*bpy), 55.7 (*C*H_2_), 55.9 (*C*H_2_), 123.4 (*C*HN_3_), 123.6 (*C*HN_3_), 124.5 (*C*H, 4-Mebpy-*C*_3_), 127.8 (*C*H, 4-Mebpy-*C*_5_), 128.2, 128.6, 129.4, 129.5, 129.6, 129.7 (all *C*H of *Ph*), 134.6 (*C_ipso_* of *Ph*), 134.9 (*C_ipso_* of *Ph*), 141.1 (*C*N_3_ of btz), 141.5 (*C*N_3_ of btz), 150.6 (*C*, 4-Mebpy-*C*_4_), 152.5 (*C*H, 4-Mebpy-*C*_6_), 158.0 ppm (*C*, 4-Mebpy-*C*_2_); HRMS (ESI): *m*/*z* calcd for [RuN_14_C_48_H_44_]^2+^ (918.02 g mol^−1^): 459.1459, found: 459.1475.

### Synthesis of [Ru(dmeobpy)(btz)_2_][PF_6_]_2_ (1 c)

[RuCl(*p*-cymene)(dmeobpy)][PF_6_] (99.9 mg, 0.16 mmol), btz (100.0 mg, 0.32 mmol, 2 equiv) and NaPF_6_ (106.3 mg, 0.63 mmol, 4 equiv) were added to a degassed solution of EtOH/H_2_O (12 mL, 3:1). The bright yellow suspension was stirred vigorously and heated at 90 °C for 18 h under N_2_. After cooling, an orange precipitate was formed, which was filtered, washed with Et_2_O (10 mL) and allowed to dry. Yield 168.1 mg (86 %).

^1^H NMR (400 MHz, CD_3_CN): *δ*=4.03 (s, 6 H, O*Me*), 5.55 (s, 8 H, btz-C*H*_2_), 6.96 (dd, ^4^*J*_HH_=2.6 Hz, ^3^*J*_HH_=6.5 Hz, 2 H, 4-OMebpy-*H*_5_), 7.14–7.18 (m, 8 H, btz-*Ph*), 7.34–7.40 (m, 12 H, btz-*Ph*), 7.72 (d, ^3^*J*_HH_=6.5 Hz, 2 H, 4-OMebpy-*H*_6_), 7.95 (d, ^4^*J*_HH_=2.7 Hz, 2 H, 4-OMebpy-*H*_3_), 8.34 (s, 2 H, C*H*N_3_), 8.35 ppm (s, 2 H, C*H*N_3_); ^13^C NMR (100.6 MHz, CD_3_CN) *δ*=55.7 (btz-*C*H_2_), 55.8 (btz-*C*H_2_), 57.2 (O*Me*), 110.6 (*C*H, *C*_3_-4-OMebpy), 113.3 (*C*H, *C*_5_-4-OMe bpy), 123.3 (*C*HN_3_), 123.5 (*C*HN_3_), 128.2, 128.6, 129.4, 129.5, 129.6, 129.7 (all *C*H of Ph), 134.6 (*C_ipso_* of *Ph*), 134.9 (*C_ipso_* of *Ph*), 141.3 (*C*N_3_), 141.6 (*C*N_3_), 153.9 (*C*H, *C*_6_-4-OMebpy), 159.4 (*C*, *C*_2_-4-OMebpy), 167.6 ppm (*C*, *C*_4_-4-OMebpy); HRMS (ESI): *m*/*z* calcd for [RuO_2_N_14_C_48_H_44_]^2+^ (950.02 g mol^−1^): 475.1401; found 475.1401.

### Synthesis of [Ru(phen)(btz)_2_][PF_6_]_2_ (1 d)

[RuCl(*p*-cymene)(phen)]PF_6_ (17.6 mg, 0.03 mmol) and btz (17.7 mg, 0.06 mmol, 2 equiv) and NaPF_6_ (20.1 mg, 0.12 mmol, 4.1 equiv) were added to a degassed solution of EtOH/H_2_O (5 mL; 3:1). The bright yellow reaction mixture was heated at 90 °C under N_2_ for 17 h. After cooling, a bright orange precipitate was formed. This was filtered and washed with Et_2_O (10 mL) and dried. Yield 22.6 mg (63 %).

^1^H NMR (400 MHz, CD_3_CN): *δ*=5.41 (s, 4 H, CH*H* of Bz), 5.62 (s, 4 H, CH*H* of Bz), 7.02 (d, *J*=7.13 Hz, 4 H, *ortho*-Ph), 7.23–7.43 (m, 16 H, *Ph*), 7.76 (dd, ^3^*J*_HH_=5.3, 8.3 Hz, 2 H, phen), 8.22 (s, 2 H, C*H*N_3_), 8.32 (s, 2 H, C*H*N_3_), 8.33 (d, ^3^*J*_HH_=5.2 Hz, 2 H, phen), 8.41 (s, 2 H, phen), 8.63 ppm (d, ^3^*J*_HH_=8.3 Hz, 2 H, phen); ^13^C NMR (100.6 MHz, CD_3_CN): *δ*=55.6 (*C*H_2_), 56.0 (*C*H_2_), 123.4 (*C*HN_3_), 123.7 (*C*HN_3_), 125.8 (*C*H, phen-*C*_5_), 128.1 (*C*H of *Ph*), 128.2 (*C*H, phen), 128.7, 129.3, 129.5, 129.6, 129.7 (all *C*H of *Ph*), 130.8 (*C*, phen), 134.5 (*C_ipso_* of *Ph*), 134.7 (*C_ipso_* of *Ph*), 137.1 (*C*H, phen), 141.3 (*C*N_3_ of btz), 141.5 (*C*N_3_ of btz), 149.1 (*C*, phen), 153.9 ppm (*C*H, phen). HRMS (ESI): *m*/*z* calcd for [RuN_14_C_48_H_40_]^+^ (913.99 g mol^−1^): 457.1302; found 457.1305.

### Synthesis of [Ru(dazf)(btz)_2_][PF_6_]_2_ (1 e)

[RuCl(*p*-cymene)(azf)]PF_6_ (49.8 mg, 0.08 mmol), btz (50.1 mg, 0.16 mmol, 2 equiv) and NaPF_6_ (57.5 mg, 0.34 mmol, 4.1 equiv) were added to a degassed solution of EtOH/H_2_O (8 mL, 3:1). The bright yellow reaction mixture was heated at 90 °C under N_2_ for 17 h. After cooling, a bright orange precipitate was formed. This was filtered and washed with Et_2_O (10 mL) and dried. Yield 45.4 mg (45 %).

^1^H NMR (400 MHz, CD_3_CN): *δ*=5.59 (s, 8 H, C*H*_2_ of Bz), 7.20–7.25 (m, 8 H, *Ph*), 7.36–7.41 (m, 12 H, *Ph*), 7.52 (dd, ^3^*J*_HH_=5.7, 7.5 Hz, 2 H, azf*-H*_5_), 7.86 (d, ^3^*J*_HH_=5.5 Hz, 2 H, azf-*H*_6_), 8.14 (d, ^3^*J*_HH_=7.6 Hz, 2 H, azf-*H*_4_), 8.38 (s, 2 H, C*H*N_3_), 8.39 (s, 2 H, C*H*N_3_); ^13^C NMR (100.6 MHz, CD_3_CN): *δ*=55.8 (*C*H_2_), 56.1 (*C*H_2_), 123.5 (*C*HN_3_), 123.8 (*C*HN_3_), 128.4 (*C*H of *Ph*), 128.7 (*C*H, azf-*C*_5_), 129.3, 129.5 (all *C*H of *Ph*), 129.5 (*C*, azf-*C*_3_), 129.6, 129.6, 129.7 (all *C*H of *Ph*), 133.4 (*C*H, azf-*C*_4_), 134.4 (*C_ipso_* of *Ph*), 134.8 (*C_ipso_* of *Ph*), 141.7 (*C*N_3_ of btz), 141.8 (*C*N_3_ of btz), 156.5 (*C*H, azf-*C*_6_), 167.7 (*C*, azf-*C*_2_), 186.8 ppm (*C*O); IR (ATR): 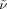
_CO_=1732 cm^−1^; HRMS (ESI): *m*/*z* calcd for [RuN_14_C_47_H_38_O]^+^ (915.97 g mol^−1^): 458.1199; found 458.1211.

### Synthesis of 1,1′-dipropyl-4,4′-bi-1,2,3-triazolyl

1-Bromopropane (1 mL, 11 mmol), sodium azide (0.79 g, 12.1 mmol) and dimethyl sulfoxide (15 mL) were stirred at RT for two hours. 1,4-Bis-(trimethylsilyl)-1,3-butadiyne (1.20 g, 6.2 mmol), CuSO_4_⋅5 H_2_O (0.36 g, 1.43 mmol), sodium ascorbate (0.87 g, 4.4 mmol), K_2_CO_3_ (1.52 g, 11 mmol) and pyridine (4.4 mL) were then added, and the orange solution was left to stir over the weekend. Water (50 mL) was then added, the solution was then filtered under vacuum, and a light brown solid was collected. The product was purified by column chromatography (5 % MeOH in dichloromethane (DCM)) to give an off-white solid (0.69 g, 51 %).

^1^H NMR (400 Hz, CDCl_3_): *δ*=8.06 (s, 2 H, CHN_3_), 4.41 (t, 4 H, NC*H*_2_CH_2_CH_3_), 2.01 (sextet, 4 H, NCH_2_C*H*_2_CH_3_), 1.02 ppm (t, 6 H, NCH_2_CH_2_C*H*_3_); ^13^C NMR (400 Hz, CDCl_3_): *δ*=11.06, 23.71, 52.10, 120.35, 140.21; HRMS (ESI): *m*/*z* calcd for [C_10_H_16_N_6_]H^+^ (221.28 g mol^−1^): 221.1509; found 221.1507.

### Synthesis of [Ru(bpy)(btz^Ph^)_2_][PF_6_]_2_ (1 a^Pr^)

[Ru(cymene)(bpy)Cl]PF_6_ (50 mg, 0.082 mmol), btz^Ph^ (46 mg, 0.16 mmol) and NaPF_6_ (60 mg, 0.18 mmol) were added to a mixture of EtOH/H_2_O (8 mL; 3:1) and heated at reflux under nitrogen in the dark overnight. The reaction mixture was cooled, and the precipitate was collected and recrystallized by using acetonitrile/diethyl ether (2:1), filtered under vacuum and washed with diethyl ether (5 mL) to give a yellow solid (62 mg, 67 %).

^1^H NMR (400 Hz, CD_3_CN): *δ*=9.01 (s, 2 H, CHN_3_), 8.99 (s, 2 H, CHN_3_), 8.50 (d, 2 H, H6-bpy), 8.35 (d, 2 H, H3-bpy), 8.11 (td, 2 H, H5-bpy)7.79- 7.81 (m, 4 H, *meta*-Ph), 7.69–7.72 (m, 4 H, *meta*-Ph), 7.58–7.62 (m, 12 H, *ortho*- and *para*-Ph), 7.48 ppm (td, 2 H, H4-bpy); ^13^C NMR (400 Hz, CD_3_CN): *δ*=121.33, 121.45, 121.51, 121.69, 123.86, 127.14, 130.57, 130.62, 130.76, 136.68, 136.80, 138.45, 141.58, 141.75, 153.92 ppm; HRMS (ESI): *m*/*z* calcd for [RuC_42_N_14_H_32_]^2+^ (833.86 g mol^−1^): 417.1000; found *m*/*z*=417.1010.

### Synthesis of [Ru(bpy)(btz^Pr^)_2_][PF_6_]_2_ (1 a^Pr^)

[Ru(cymene)(bpy)Cl]PF_6_ (50 mg, 0.082 mmol), btz^Pr^ (36 mg, 0.16 mmol) and NaPF_6_ (60 mg, 0.18 mmol) were added to a mixture of EtOH/H_2_O (8 mL; 3:1) and heated at reflux under nitrogen in the dark overnight. The resultant orange solution was cooled, and the precipitate was filtered to give a yellow/orange solid (51 mg, 63 %).

^1^H NMR (400 Hz, CD_3_CN): *δ*=8.43 (d, 2 H, H6-bpy), 8.40 (s, 2 H, CHN_3_), 8.38 (s, 2 H, CHN_3_), 8.01–8.07 (m, 4 H, H3-bpy and H5-bpy), 7.42 (t, 2 H, H4-bpy), 4.36 (t, 4 H, NC*H*_2_CH_2_CH_3_), 4.31 (t, 4 H, NC*H*_2_CH_2_CH_3_), 1.76–1.91 (m, 8 H, NCH_2_CH_2_CH_3_ and NCH_2_C*H*_2_CH_3_), 0.83 (t, 6 H, NCH_2_CH_2_C*H*_3_), 0.78 ppm (t, 6 H, NCH_2_CH_2_C*H*_3_); ^13^C NMR (400 Hz, CD_3_CN): *δ*=10.25, 10.51, 23.51, 23.60, 53.99, 54.10, 122.93, 123.25, 123.67, 126.96, 137.91, 140.91, 141.17, 153.40, 158.54 ppm; HRMS (ESI): *m*/*z* calcd for [RuC_30_N_14_H_30_]^2+^ (687.72 g mol^−1^): 349.1311; found 349.1318.

## Supporting Information

As a service to our authors and readers, this journal provides supporting information supplied by the authors. Such materials are peer reviewed and may be re-organized for online delivery, but are not copy-edited or typeset. Technical support issues arising from supporting information (other than missing files) should be addressed to the authors.

miscellaneous_information
