# Supplementary material for: Photochemistry of RuII 4,4′-Bi-1,2,3-triazolyl (btz) Complexes: Crystallographic Characterization of the Photoreactive Ligand-Loss Intermediate trans-[Ru(bpy)(κ2-btz)(κ1-btz)(NCMe)]2+
Source: Chemistry. 2014 May 30;20(27):8467–76. doi: 10.1002/chem.201402354 (PMC4506527; doi:10.1002/chem.201402354)
Supplement: Supplementary file 1 — miscellaneous_information [file chem0020-8467-sd1.pdf]

CHEMISTRY
A European Journal

Supporting Information

© Copyright Wiley-VCH Verlag GmbH & Co. KGaA, 69451 Weinheim, 2014

Photochemistry of Ru^{II} 4,4′-Bi-1,2,3-triazolyl (btz) Complexes: Crystallographic Characterization of the Photoreactive Ligand-Loss Intermediate *trans*-[Ru(bpy)(κ^2-btz)(κ^1-btz)(NCMe)]$^{2+}$

Christine E. Welby, Georgina K. Armitage, Harry Bartley, Aaron Wilkinson, Alessandro Sinopoli, Baljinder S. Uppal, Craig R. Rice, and Paul I. P. Elliott*[a]

chem_201402354_sm_miscellaneous_information.pdf

Photochemical conversion of $[Ru(bpy)(btz)_2][PF_6]_2$ (**1a)** in acetonitrile 2

Photochemical conversion of $[Ru(dmbpy)(btz)_2][PF_6]_2$ (**1b**) in acetonitrile 3

Photochemical conversion of $[Ru(dmeobpy)(btz)_2][PF_6]_2$ (**1c**) in acetonitrile 4

Photochemical conversion of $[Ru(phen)(btz)_2][PF_6]_2$ (**1d**) in acetonitrile 5

Photochemical conversion of $[Ru(bpy)(btz^{Ph})_2][PF_6]_2$ (**1a^{Ph}**) in acetonitrile 6

Photochemical conversion of $[Ru(bpy)(btz^{Pr})_2][PF_6]_2$ (**1a^{Pr}**) in acetonitrile 7

DFT atomic coordinates for *cis*/*trans*-$[Ru(bpy)(btz)(NCMe)_2]^{2+}$

and *cis*/*trans*-$[Ru(bpy)_2(NCMe)_2]^{2+}$ 8

Table S1. X-ray Crystallographic data for complexes **1d**, **1e**, **2a** and **3d**. 16

Photochemical conversion of $[Ru(bpy)(btz)_2][PF_6]_2$ (1a) in acetonitrile

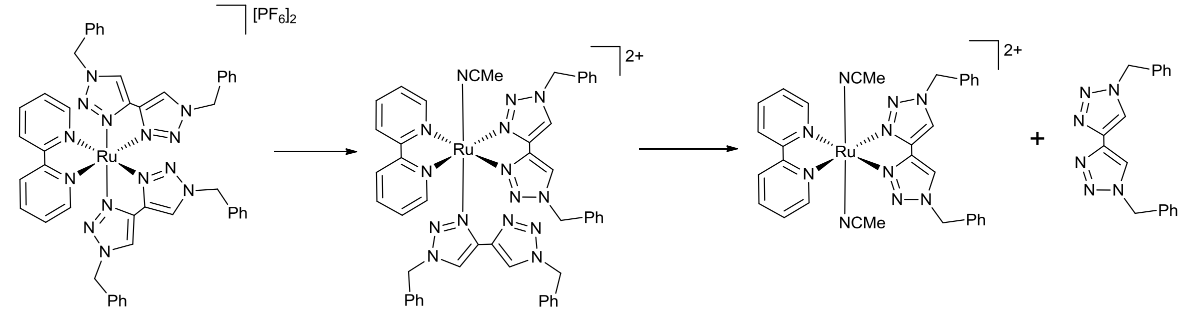

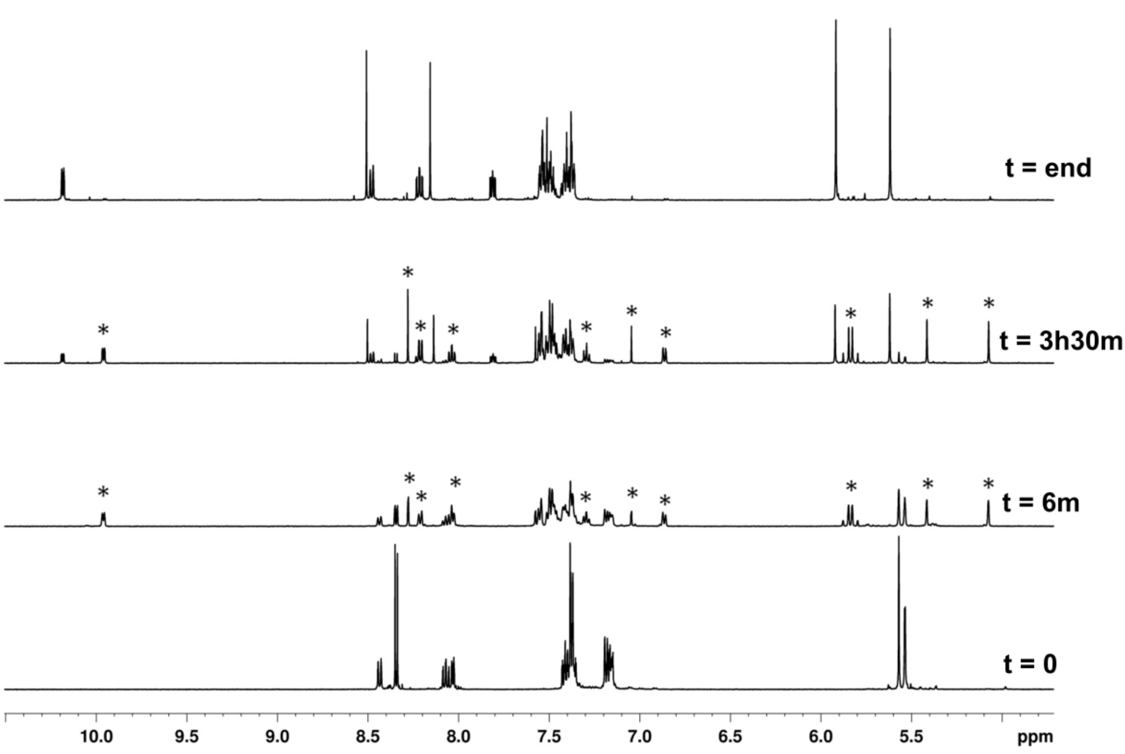

Figure S1. ^{1}H NMR spectra recorded during photochemical conversion of **1a** in d_3-acetonitrile (* signals for **2a**)

***Trans*-$[Ru(bpy)(\kappa^1\text{-btz})(\kappa^2\text{-btz})(MeCN)]^{2+}$ 2a:**

^{1}H NMR (500 MHz) CD_3CN δ_H 5.07 (s, 2H, CH_2 of κ^1-btz), 5.40 (s, 2H, CH_2 of κ^1-btz), 5.81 (d*, $^2J_{HH}$ = 15.0 Hz, 2H, CH_2 of κ^2-btz), 5.87 (d*, $^2J_{HH}$ = 15.0 Hz, 2H, CH_2 of κ^2-btz), 6.86 (d, $^3J_{HH}$ = 7.6 Hz, 2H, Ph of κ^1-btz), 7.05 (s, 1H, CHN$_3$ of κ^1-btz), 7.29 (t, $^3J_{HH}$ = 7.5 Hz, 2H, Ph of κ^1-btz), 7.34 – 7.57 (m, 18H, Ph of κ^2-btz {10H} + Ph of κ^1-btz {6H} + H_5-bpy {2H}), 7.58 (s, 1H, CHN$_3$ of κ^1-btz), 8.07 (t, $^3J_{HH}$ = 7.8 Hz, 2H, H_4-bpy), 8.21 (d, $^3J_{HH}$ = 8.0 Hz, 2H, H_3-bpy), 8.29 (s, 2H, CHN$_3$ of κ^2-btz), 9.96 (d, $^3J_{HH}$ = 5.6 Hz, 2H, H_6-bpy);); ^{13}C NMR (100.6 MHz) CD_3CN δ_C 53.6 (CH$_2$ of κ^1-btz), 54.7 (CH$_2$ of κ^1-btz), 55.5 (CH$_2$ of κ^2-btz), 122.4 (CHN$_3$ of κ^2-btz), 122.6 (CH, bpy-C_3), 125.2 (CHN$_3$ of κ^1-btz), 125.9 (CH, bpy-C_5), 126.5 (CH of Ph), 127.7 (CHN$_3$ of κ^1-btz), 128.0, 128.3, 128.5, 128.6, 128.9, 129.1, 129.1, 129.2 (CH of Ph), 133.7, (C, *ipso*-Ph of κ^1-btz), 134.0 (CN$_3$ of κ^1-btz), 134.3 (C, *ipso*-Ph of κ^2-btz), 135.2 (C, *ipso*-Ph of κ^1-btz), 137.7 (CH, bpy-C_4), 141.3 (CN$_3$ of κ^2-btz), 142.6 (CN$_3$ of κ^1-btz), 155.6 (CH, bpy-C_6), 158.7 (C, bpy-C_2).

***trans*-$[Ru(bpy)(btz)(MeCN)_2]^{2+}$ 3a:**

^{1}H NMR (400 MHz) CD_3CN δ_H 5.91 (s, 4H, CH_2), 7.46 – 7.57 (m, 10H, *Ph*), 7.80 (t, $^3J_{HH}$ = 6.6 Hz, 2H, H_5-bpy), 8.21 (t, $^3J_{HH}$ = 7.9 Hz, 2H, H_4-bpy), 8.48 (d, $^3J_{HH}$ = 8.1 Hz, 2H, H_3-bpy), 8.50 (s, 2H, CHN$_3$), 10.2 (d, $^3J_{HH}$ = 5.6 Hz, 2H, H_6-bpy); ^{13}C NMR (100.6 MHz) CD_3CN δ_C 56.2 (CH$_2$), 123.5 (CHN$_3$), 123.7 (CH, C$_3$-bpy), 127.2 (CH, C$_5$-bpy), 129.1, 129.7, 129.8 (CH, Ph), 134.7 (*C-ipso*, Ph), 139.0 (CH, C$_4$-bpy), 141.8 (C, CN$_3$), 156.1 (CH, C$_6$-bpy), 159.1 (C, C$_2$-bpy)

Photochemical conversion of $[Ru(dmbpy)(btz)_2][PF_6]_2$ (1b) in acetonitrile

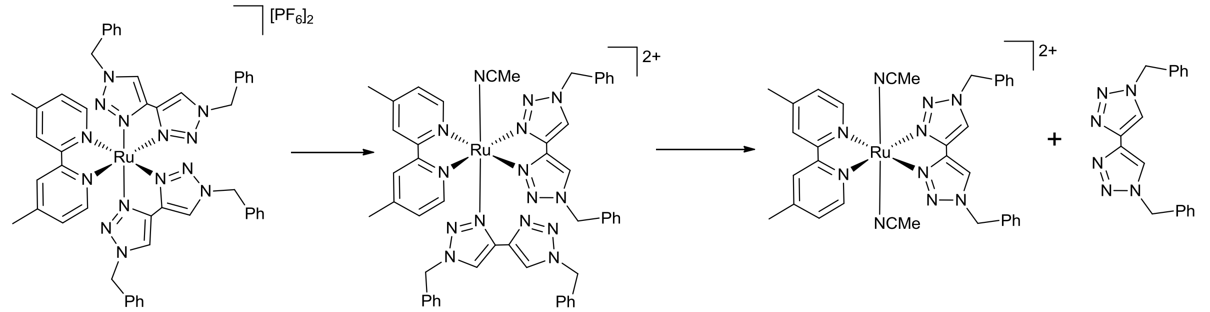

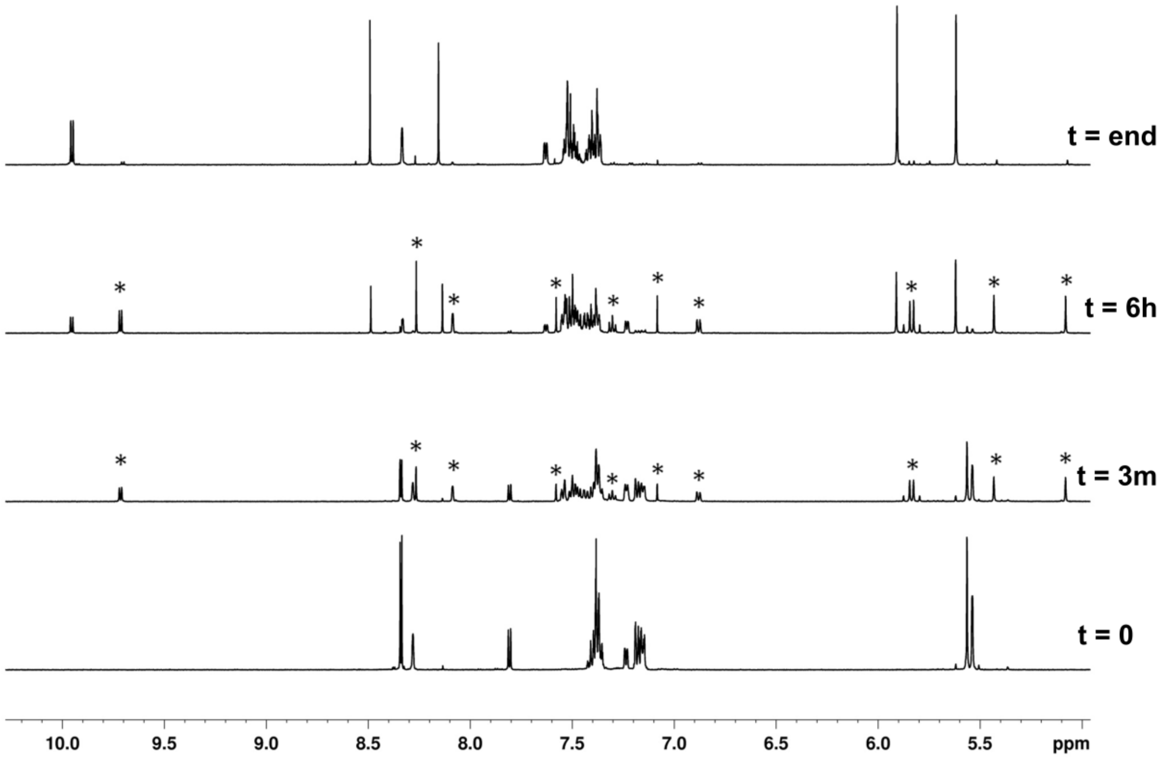

Figure S3. ^{1}H NMR spectra recorded during photochemical conversion of **1b** in d_3-acetonitrile (* signals for **2b**)

***Trans*-[Ru(4-Mebpy)(κ^1-btz)(κ^2-btz)(MeCN)]$^{2+}$ 3b:**

^{1}H NMR (500 MHz) CD_3CN δ_H 2.60 (s, 6H, 4-*Me*bpy), 5.07 (s, 2H, CH_2 of κ^1-btz), 5.42 (s, 2H, CH_2 of κ^1-btz), 5.81 (d*, $^2J_{HH}$ = 15.0 Hz, 2H, CH_2 of κ^2-btz), 5.87 (d*, $^2J_{HH}$ = 15.0 Hz, 2H, CH_2 of κ^2-btz), 6.88 (d, $^3J_{HH}$ = 7.3 Hz, 2H, Ph of κ^1-btz), 7.08 (s, 1H, C*H*N_3 of κ^1-btz), 7.30 (t, $^3J_{HH}$ = 7.8 Hz, 2H, Ph of κ^1-btz), 7.34 – 7.57 (m, 18H, Ph of κ^2-btz {10H} + Ph of κ^1-btz {6H} + H_5-4-Mebpy {2H}), 7.59 (s, 1H, C*H*N_3 of κ^1-btz), 8.09 (br s, 2H, H_3-4-Mebpy), 8.27 (s, 2H, C*H*N_3 of κ^2-btz), 9.70 (d, $^3J_{HH}$ = 5.8 Hz, 2H, H_6-4-Mebpy); ^{13}C NMR (125.8 MHz) CD_3CN δ_C 25.6 (*C*H_3, 4-*Me*bpy), 58.9 (*C*H_2 of κ^1-btz), 60.0 (*C*H_2 of κ^1-btz), 60.8 (*C*H_2 of κ^2-btz), 127.7 (*C*HN_3 of κ^2-btz), 128.3 (*C*H, 4-Mebpy-C_5), 128.6 (*C*H, 4-Mebpy-C_3), 130.7 (*C*HN_3 of κ^1-btz), 132.0 (*C*H of Ph), 133.0 (*C*HN_3 of κ^1-btz), 133.6, 133.8, 133.9, 134.1, 134.2, 134.4, 134.5, 134.5 (*C*H of Ph), 139.1, (*C*, *ipso*-Ph of κ^1-btz), 139.3 (*C*N_3 of κ^1-btz), 139.7 (*C*, *ipso*-Ph of κ^2-btz), 140.6 (*C*, *ipso*-Ph of κ^1-btz), 146.6 (*C*N_3 of κ^2-btz), 147.9 (*C*N_3 of κ^1-btz), 155.3 (*C*, 4-Mebpy-C_4), 160.0 (*C*H, 4-Mebpy-C_6), 163.6 (*C*, 4-Mebpy-C_2).

***trans*-[Ru(4-Mebpy)(btz)(MeCN)$_2$]$^{2+}$ 4b:**

^{1}H NMR (400 MHz) CD_3CN δ_H 2.67 (s, 6H, 4-*Me*bpy), 5.90 (s, 4H, CH_2), 7.44 – 7.55 (m, 10H, *Ph*), 7.63 (d, $^3J_{HH}$ = 5.7 Hz, 2H, H_5-4-Mebpy), 8.33 (br s, 2H, H_3-4-Mebpy), 8.48 (s, 2H, C*H*N_3), 9.95 (d, $^3J_{HH}$ = 5.8 Hz, 2H, H_6-4-Mebpy); ^{13}C NMR (100.6 MHz) CD_3CN δ_C 20.9 (*C*H_3, 4-*Me*bpy), 56.2 (*C*H_2), 123.4 (*C*HN_3), 124.4 (*C*H, C_3-4-Mebpy), 127.9 (*C*H, C_5-4-Mebpy), 129.1, 129.7, 129.8 (all *C*H of *Ph*), 134.7 (C_{ipso} of *Ph*), 141.9 (*C*N_3 of btz), 151.4 (*C*, C_4-4-Mebpy), 155.2 (*C*H, C_6-4-Mebpy), 158.6 (*C*, C_2-4-Mebpy).

Photochemical conversion of [Ru(dmeobpy)(btz)$_2$][PF$_6$]$_2$ (1c) in acetonitrile

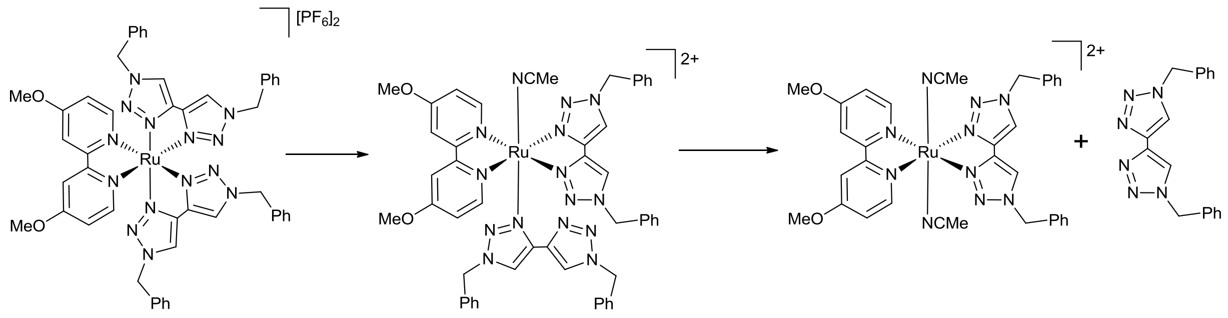

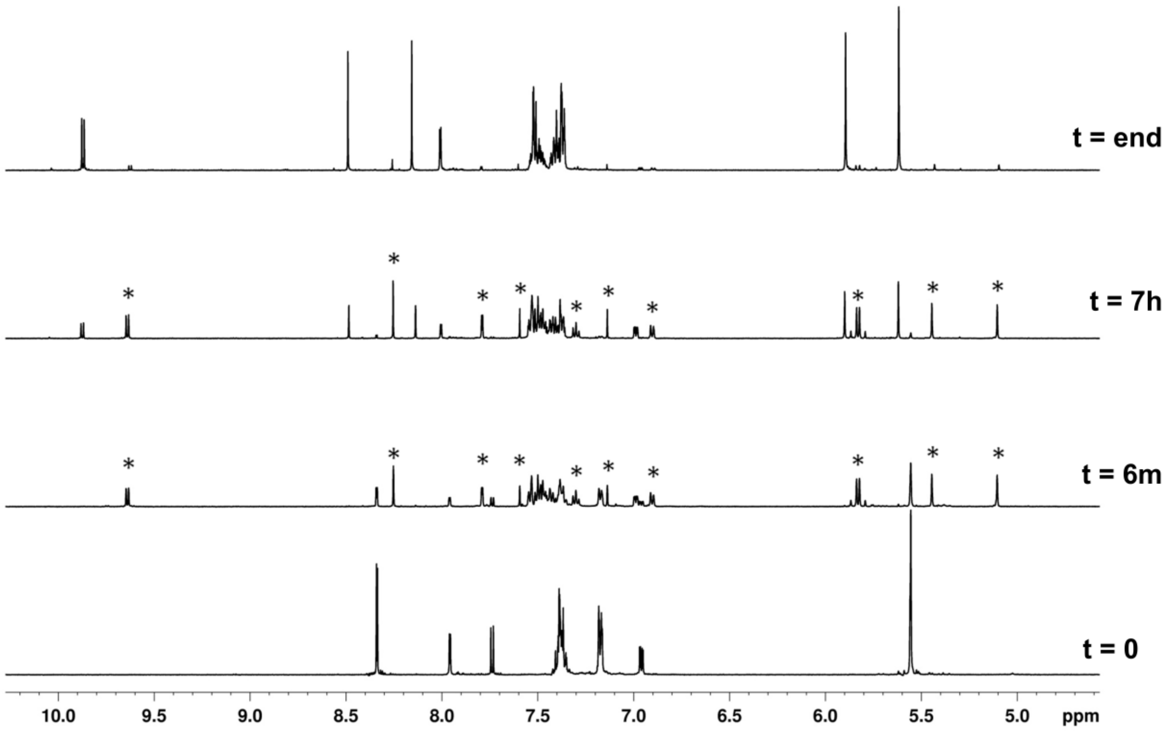

Figure S4. ^{1}H NMR spectra recorded during photochemical conversion of **1c** in d$_3$-acetonitrile (* signals for **2c**)

***Trans*-[Ru(4-OMebpy)(κ^1-btz)(κ^2-btz)(MeCN)]$^{2+}$ 3c:**

^{1}H NMR (400 MHz) CD$_3$CN δ_H 4.06 (s, 6H, 4-O*Me*bpy), 5.09 (s, 2H, C*H*$_2$ of κ^1-btz), 5.43 (s, 2H, C*H*$_2$ of κ^1-btz), 5.80 (d*, $^2J_{HH}$ = 15.0 Hz, 2H, C*H*$_2$ of κ^2-btz), 5.86 (d*, $^2J_{HH}$ = 15.0 Hz, 2H, C*H*$_2$ of κ^2-btz), 6.90 (d, $^3J_{HH}$ = 7.7 Hz, 2H, Ph of κ^1-btz), 6.96 (dd, $^3J_{HH}$ = 6.5 Hz, $^2J_{HH}$ = 2.7 Hz, 2H, H_5-4-OMebpy), 7.13 (s, 1H, C*H*N$_3$ of κ^1-btz), 7.30 (t, $^3J_{HH}$ = 7.5 Hz, 3H, Ph of κ^1-btz), 7.45 – 7.54 (m, 15H, Ph of κ^2-btz {10H} + Ph of κ^1-btz {5H}), 7.60 (s, 1H, C*H*N$_3$ of κ^1-btz), 7.79 (d, $^3J_{HH}$ = 2.7 Hz 2H, H_3-4-OMebpy), 8.25 (s, 2H, C*H*N$_3$ of κ^2-btz), 9.62 (d, $^3J_{HH}$ = 6.5 Hz, 2H, H_6-4-OMebpy).

***trans*-[Ru(4-OMebpy)(btz)(MeCN)$_2$]$^{2+}$ 4c:**

^{1}H NMR (400 MHz) CD$_3$CN δ_H 4.12 (s, 6H, 4-O*Me*bpy), 5.89 (s, 4H, C*H*$_2$), 7.31 – 7.44 (m, 12H, *Ph* {10H of free btz} + H_5-4-OMebpy {2H}), 7.44 – 7.55 (m, 10H, *Ph*), 8.00 (br s, 2H, H_3-4-OMebpy), 8.49 (s, 2H, C*H*N$_3$), 9.87 (d, $^3J_{HH}$ = 6.4 Hz, 2H, H_6-4-Mebpy); ^{13}C NMR (100.6 MHz) CD$_3$CN δ_C 56.1 (btz-*C*H$_2$), 57.2 (O*Me*), 110.6 (*C*H, C_3-4-OMebpy), 113.3 (*C*H, C_5-4-OMe bpy), 123.4 (*C*HN$_3$), 129.1, 129.7, 129.8 (all *C*H of Ph), 134.8 (C_{ipso} of *Ph*), 141.9 (*C*N$_3$), 156.5 (*C*H, C_6-4-OMebpy), 160.0 (*C*, C_2-4-OMebpy), 168.3 (*C*, C_4-4-OMebpy).

Photochemical conversion of $[Ru(phen)(btz)_2][PF_6]_2$ (1d) in acetonitrile

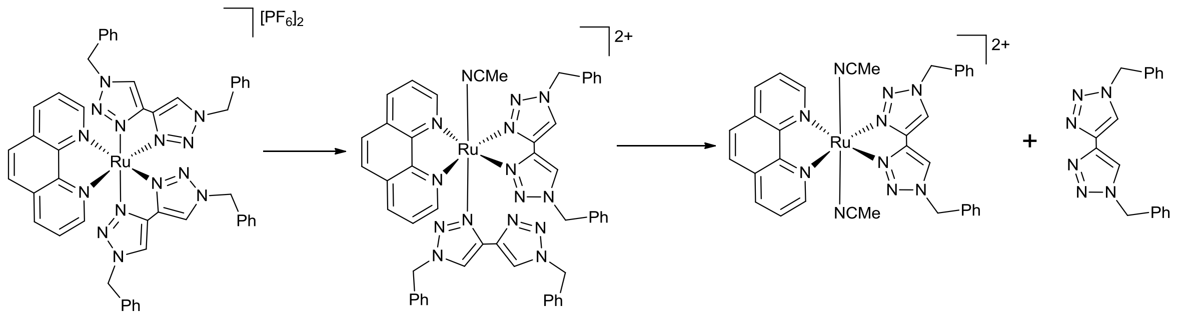

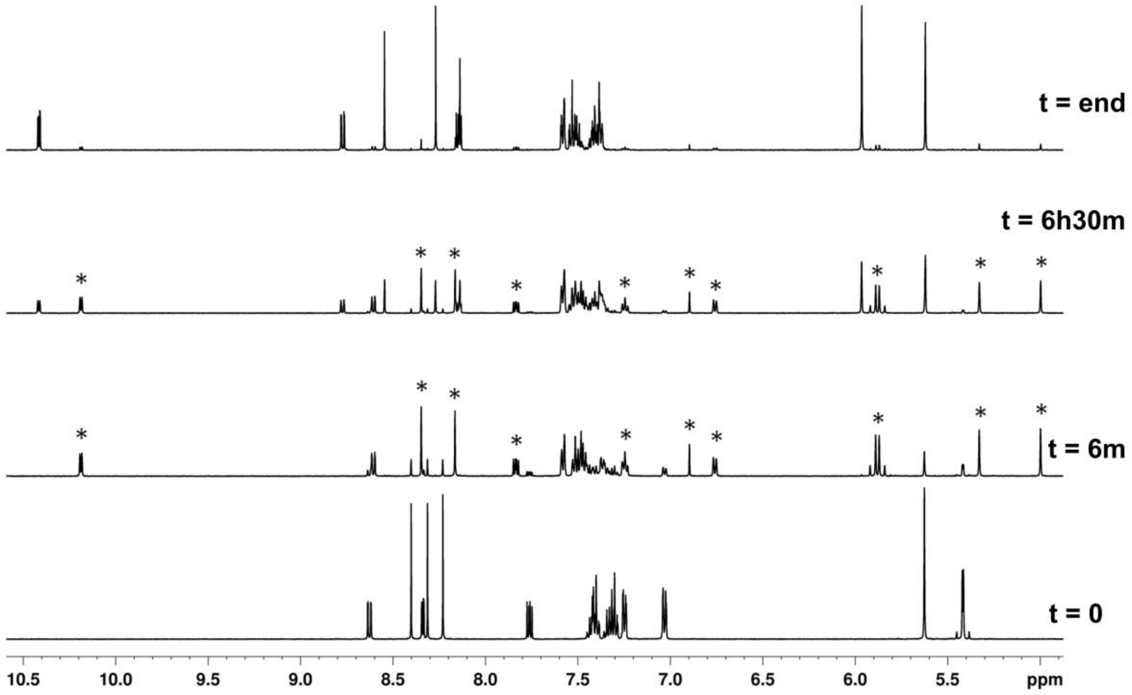

Figure S5. ^{1}H NMR spectra recorded during photochemical conversion of **1d** in d_3-acetonitrile (* signals for **2d**)

$[Ru(phen)(\kappa^1\text{-btz})(\kappa^2\text{-btz})(MeCN)]^{2+}$ 3d:

^{1}H NMR (400 MHz) CD_3CN δ_H 4.99 (s, 2H, CH_2 of κ^1-btz), 5.31 (s, 2H, CH_2 of κ^1-btz), 5.85 (d*, $^2J_{HH}$ = 14.9 Hz, 2H, CH_2 of κ^2-btz), 5.91 (d*, $^2J_{HH}$ = 15.0 Hz, 2H, CH_2 of κ^2-btz), 6.75 (d, $^3J_{HH}$ = 7.6 Hz, 2H, Ph of κ^1-btz), 6.88 (s, 1H, C*H*N_3 of κ^1-btz), 7.19 – 7.60 (m, 19H, Ph of κ^2-btz {18H} + C*H*N_3 of κ^1-btz {1H}), 7.82 (dd, $^3J_{HH}$ = 5.2, 8.4 Hz, 2H, phen-H_5), 8.16 (s, 2H, phen-$H_{3''}$), 8.35 (s, 2H, C*H*N_3 of κ^2-btz), 8.61 (d, $^3J_{HH}$ = 8.1 Hz, 2H, phen-H_4), 10.2 (d, $^3J_{HH}$ = 5.1 Hz, 2H, phen-H_6); ^{13}C NMR (100.6 MHz) CD_3CN δ_C 54.0 (*C*H_2 of κ^1-btz), 55.1 (*C*H_2 of κ^1-btz), 56.1 (*C*H_2 of κ^2-btz), 123.1 (*C*HN_3 of κ^2-btz), 125.5 (*C*HN_3 of κ^1-btz), 125.6 (*C*H, phen-C_5), 127.8 (*C*H, phen-$C_{3''}$), 128.3 (*C*HN_3 of κ^1-btz), 128.7, 128.8, 129.0, 129.1, 129.4, 129.4 (*C*H of Ph), 129.5 (*C*N_3 of κ^1-btz), 129.6, 129.7, 129.8 (*C*H of Ph), 130.5 (*C*, phen-C_3), 134.1 (*C*, *ipso*-Ph of κ^1-btz), 134.9 (*C*, *ipso*-Ph of κ^2-btz), 135.7 (*C*, *ipso*-Ph of κ^1-btz), 137.1 (*C*H, phen-C_4), 142.1 (*C*N_3 of κ^2-btz), 143.0 (*C*N_3 of κ^2-btz), 150.0 (*C*, phen-C_2), 156.6 (*C*H, phen-C_6).

***trans*-$[Ru(phen)(btz)(MeCN)_2]^{2+}$ 4d:**

^{1}H NMR (500 MHz) CD_3CN δ_H 5.96 (s, 4H, CH_2), 7.48 – 7.54 (m, 10H, *Ph*), 8.15 (dd, $^3J_{HH}$ = 5.2, 8.1 Hz, 2H, phen-H_5), 8.26 (s, 2H, phen-$H_{3''}$), 8.55 (s, 2H, C*H*N_3), 8.77 (d, $^3J_{HH}$ = 8.1 Hz, 2H, phen-H_4), 10.4 (d, $^3J_{HH}$ = 5.3 Hz, 2H, phen-H_6); ^{13}C NMR (125.8 MHz) CD_3CN δ_C 56.7 (*C*H_2), 124.0 (*C*HN_3), 126.4 (*C*H, phen-C_5), 128.5 (*C*H, phen-$C_{3''}$), 129.5, 130.1, 130.2 (*C*H, Ph), 131.3 (*C*, phen-C_2), 135.2 (*C*, *ipso*-Ph), 138.2 (*C*H, phen-C_4), 142.5 (*C*, *C*N_3), 150.3 (*C*, phen-C_2), 156.9 (*C*H, phen-C_6).

Photochemical conversion of $[Ru(bpy)(btz^{Ph})_2][PF_6]_2$ ($1a^{Ph}$) in acetonitrile

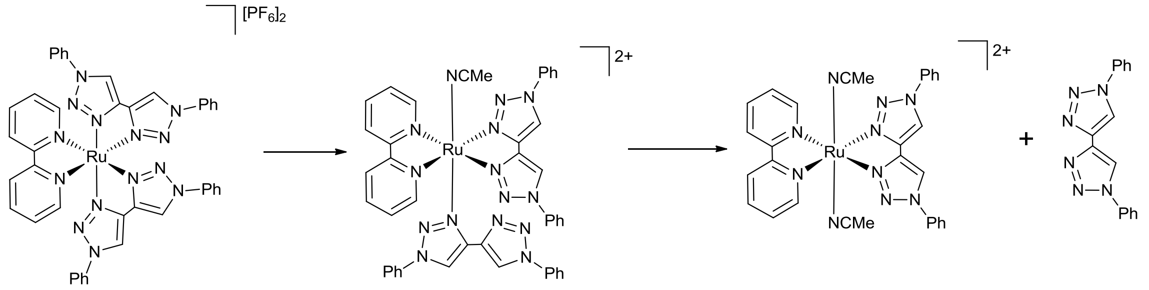

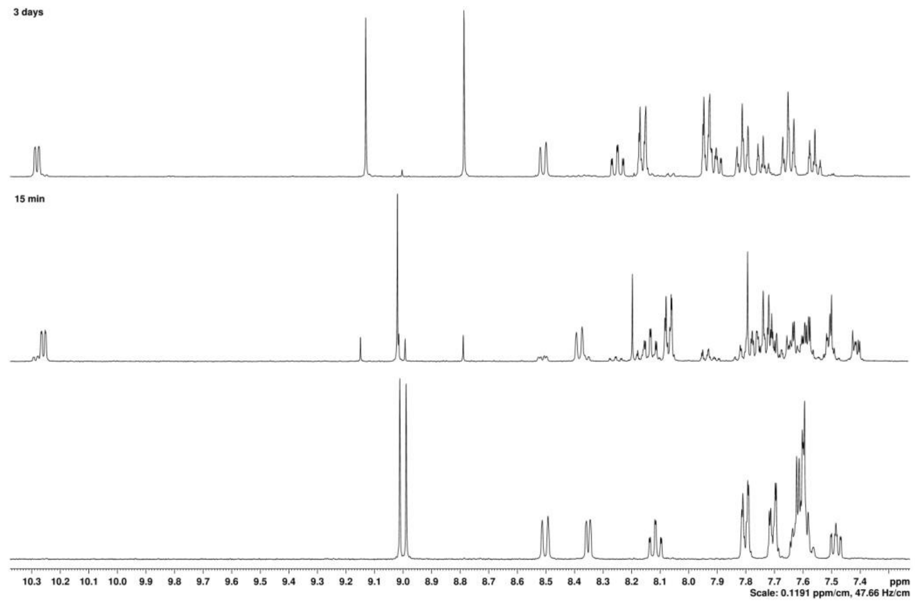

Photochemical conversion of [Ru(bpy)(btz^{Pr})$_2$][PF$_6$]$_2$ (1a^{Pr}) in acetonitrile

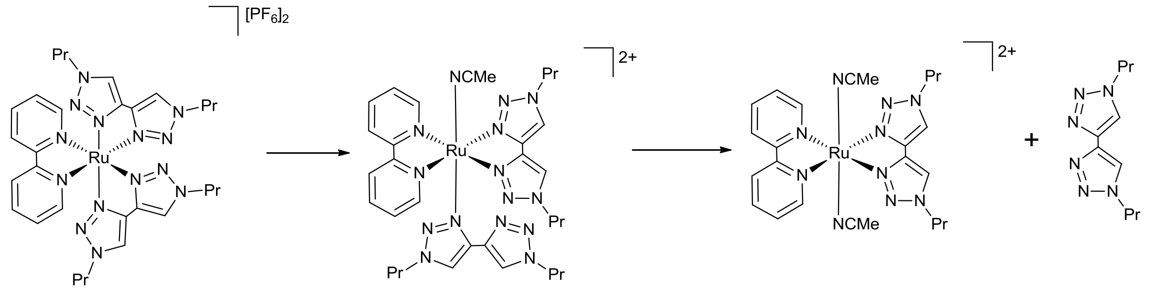

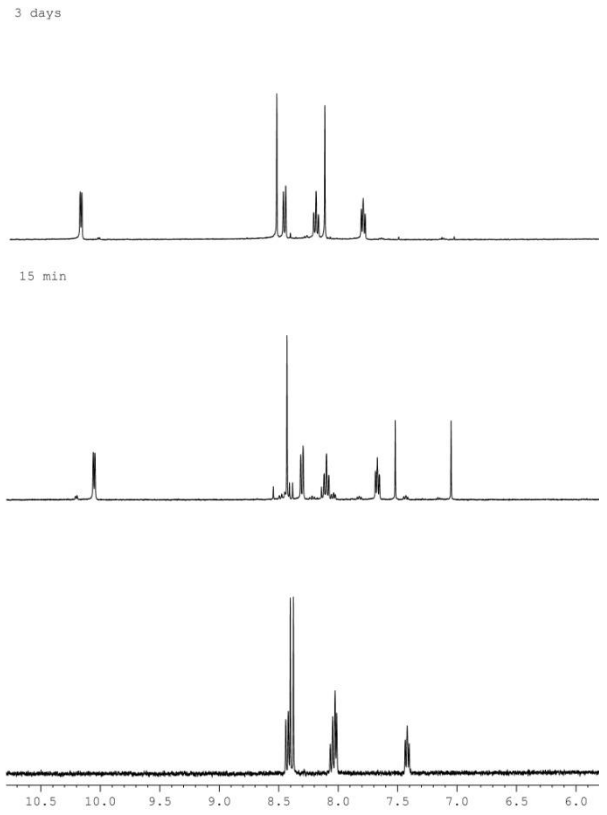

Figure S7. ^{1}H NMR spectra recorded during photochemical conversion of **1a^{Pr}** in d$_3$-acetonitrile.

Atomic coordinates for DFT optimised geometries

Trans-$[Ru(bpy)(btz)(NCMe)_2]^{2+}$

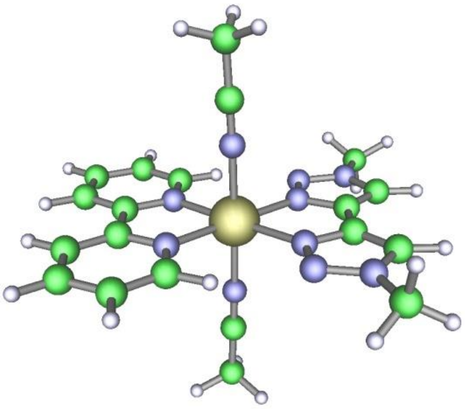

53
geometry

C	3.02063148	-3.77418300	-0.66144602
C	1.69170112	-3.95659097	-0.29778676
C	0.86086763	-2.84844197	-0.13369893
N	1.33598598	-1.58833072	-0.32176487
C	2.62061674	-1.41545542	-0.67297167
C	3.49309108	-2.48071662	-0.85356602
C	-1.23067978	-4.13879612	0.48313933
C	-2.57720831	-4.12262606	0.82671121
C	-3.23191489	-2.89972878	0.92204364
C	-2.51623515	-1.73602328	0.67223248
N	-1.21510247	-1.74817681	0.34045215
C	-0.56395870	-2.93768765	0.24129654
N	0.45049487	0.01706714	1.94522164
N	2.35478924	1.90801319	-0.68957532
N	2.52938940	3.22866154	-0.76745053
C	1.38069955	3.89855005	-0.50506839
C	0.45202693	2.91295250	-0.25672079
N	1.10196347	1.71264397	-0.38346017
N	-2.70172758	1.58915848	0.54808722
N	-3.05029694	2.87646035	0.58829357
C	-2.00080115	3.68511424	0.30240725
C	-0.94938758	2.82463575	0.07890812
N	-1.43419716	1.55298623	0.24310823
C	3.85907400	3.78067379	-1.04298476

C	-4.44074856	3.25384440	0.85758234
Ru	-0.05220341	-0.04498473	-0.03388263
H	3.67279293	-4.62976180	-0.79293351
H	1.30946227	-4.95710275	-0.14584535
H	2.94182012	-0.39103037	-0.80861666
H	4.52023413	-2.28981413	-1.13900657
H	-0.70900115	-5.08343317	0.40671572
H	-3.10291629	-5.05123064	1.01636067
H	-4.28025332	-2.83868759	1.18706969
H	-2.97999194	-0.76034524	0.73578960
H	1.32179403	4.97445864	-0.51544774
H	-2.08613892	4.75915288	0.28279816
H	3.74594463	4.74432965	-1.53542315
H	4.38675069	3.09374639	-1.69993472
H	4.41181121	3.90208830	-0.11127022
H	-4.45493102	4.21439970	1.36879609
H	-4.88229209	2.49278066	1.49595091
H	-4.99630383	3.32185199	-0.07790416
C	0.73576683	0.01666812	3.06006586
C	1.09478605	0.00800897	4.46870554
H	0.20490188	0.14517159	5.08665314
H	1.55969777	-0.94451093	4.73196595
H	1.80065537	0.81232013	4.68632306
N	-0.55596904	-0.15176687	-2.01119633
C	-0.84159884	-0.24615206	-3.12196416
C	-1.20391041	-0.36969926	-4.52441227
H	-2.28915804	-0.33018673	-4.63968210
H	-0.75970198	0.44211394	-5.10406864
H	-0.84442289	-1.32023800	-4.92425744

***Cis*-[Ru(bpy)(btz)(NCMe)$_2$]$^{2+}$**

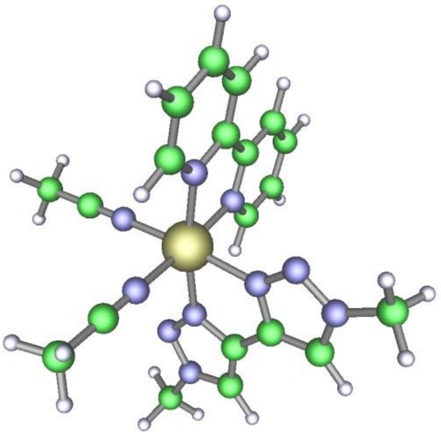

53
geometry

C	-1.84845869	2.33260892	3.44395683
C	-1.38342501	2.98127272	2.30702035
C	-0.70588221	2.25959622	1.32361696
N	-0.49976854	0.92181772	1.46061238
C	-0.94113612	0.30232810	2.56797521
C	-1.61879460	0.96737693	3.57972523
C	-0.28309547	4.21268864	-0.23013272
C	0.27891407	4.69483376	-1.40558712
C	0.96269370	3.81151428	-2.23376815
C	1.05588312	2.47967215	-1.85579923
N	0.51486878	2.00751717	-0.72192765
C	-0.15345296	2.86294529	0.09682384
N	0.88053252	-2.83164970	1.25741015
N	0.20206736	-3.98201113	1.23754237
C	-0.90309716	-3.89956456	0.45633486
C	-0.89380700	-2.61003644	-0.02536452
N	0.22035700	-2.00004349	0.49818289
N	-2.04952609	0.17001196	-1.84590238
N	-3.03249990	-0.66783841	-2.18169399
C	-2.87188387	-1.88634452	-1.60843548
C	-1.71004237	-1.77786028	-0.87839199
N	-1.24953099	-0.49743191	-1.06144355
C	0.71614423	-5.14844774	1.96094636
C	-4.13324955	-0.20292902	-3.03059368
Ru	0.54118525	0.00759445	-0.10465332
H	-2.37449736	2.88672405	4.21270362
H	-1.54533180	4.04476244	2.19342566
H	-0.73040644	-0.75647337	2.63489482
H	-1.95275390	0.42158113	4.45355558
H	-0.81908991	4.88758873	0.42367267

H	0.18300853	5.74184500	-1.66819506
H	1.41880566	4.14080392	-3.15915320
H	1.57376348	1.75633187	-2.47014907
H	-1.57619544	-4.72906836	0.31468717
H	-3.56816887	-2.69460208	-1.76073367
H	-0.12229574	-5.75700241	2.29378621
H	1.27799423	-4.79412123	2.82140228
H	1.36577420	-5.73398042	1.31031268
H	-4.41667878	-0.99793119	-3.71811924
H	-3.78136671	0.65974132	-3.59023842
H	-4.98639059	0.07835712	-2.41306670
N	2.29081587	0.28691656	0.92535946
C	3.26837063	0.42202290	1.51621893
C	4.50263232	0.58595891	2.26645626
N	1.61376495	-0.73141525	-1.70710092
C	2.22862037	-1.17052819	-2.57581490
C	3.00935900	-1.71858511	-3.67350580
H	5.27958315	1.01567965	1.63063932
H	4.34142246	1.25138628	3.11720080
H	4.84828757	-0.38087594	2.63844132
H	4.07604167	-1.58096103	-3.48392619
H	2.80993556	-2.78667269	-3.78286084
H	2.75280518	-1.21874764	-4.61002182

***Trans*-[Ru(bpy)$_2$(NCMe)$_2$]$^{2+}$**

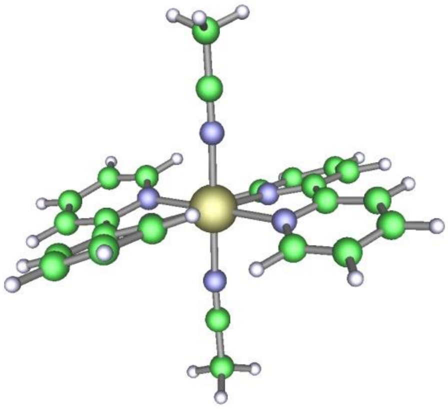

53
geometry

C	2.87105070	-3.98696725	-0.00860583
C	1.49814799	-4.04542147	0.19288230
C	0.73287202	-2.88183479	0.10239100
N	1.31515360	-1.67392449	-0.12378961
C	2.63284152	-1.63909869	-0.37846913
C	3.44529663	-2.76352069	-0.33492174
C	-1.51470124	-4.03849531	0.14883569
C	-2.88240292	-3.97108303	-0.08385873
C	-3.44296444	-2.74189949	-0.41332605
C	-2.62350207	-1.62186214	-0.43074680
N	-1.31222889	-1.66596978	-0.14562023
C	-0.74146315	-2.87809843	0.08583572
N	-0.00654789	-0.17002450	2.06375316
Ru	0.00286940	-0.00030745	0.02547614
H	3.47240226	-4.88608319	0.05650589
H	1.02850312	-4.99921860	0.39272272
H	3.04197680	-0.67524330	-0.64348811
H	4.50209255	-2.67279133	-0.55385913
H	-1.05452751	-4.99645665	0.35034410
H	-3.48966819	-4.86764442	-0.03942099
H	-4.49410056	-2.64427942	-0.65564571
H	-3.02139949	-0.65377904	-0.69861598
C	-0.01065000	-0.22128429	3.21401195
C	-0.01505589	-0.27962416	4.66724735
H	-1.01582174	-0.07197074	5.05258073
H	0.29147585	-1.27123660	5.00681976
H	0.67651099	0.45960434	5.07778418
N	0.01496200	0.16180869	-2.01419667
C	0.02489314	0.19424333	-3.16500918
C	0.03852991	0.22466414	-4.61873891
H	-0.96831637	0.39743106	-5.00483914

H	0.69154927	1.02389938	-4.97527805
H	0.40436407	-0.72635854	-5.01233039
C	2.87291626	3.97703968	-0.05939160
C	1.50547944	4.02207707	-0.30024193
C	0.73631382	2.86760387	-0.14792866
N	1.31254425	1.67615089	0.16647106
C	2.62114270	1.65991593	0.46608712
C	3.43523870	2.78046951	0.37186332
C	-1.51143746	4.02016880	-0.27793127
C	-2.87703201	3.96956124	-0.02782231
C	-3.43409471	2.76709180	0.39403165
C	-2.61660736	1.64792643	0.47255481
N	-1.31013675	1.67043425	0.16436917
C	-0.73869136	2.86563754	-0.14347004
H	3.47800337	4.86899470	-0.17315751
H	1.04233694	4.95966472	-0.57756233
H	3.02085387	0.71589661	0.80794116
H	4.48424991	2.70773579	0.63154220
H	-1.05234523	4.96207418	-0.54666910
H	-3.48473968	4.86129976	-0.12769159
H	-4.48117304	2.68936522	0.65996079
H	-3.01171772	0.69945713	0.80799772

Cis-[Ru(bpy)$_2$(NCMe)$_2$]$^{2+}$

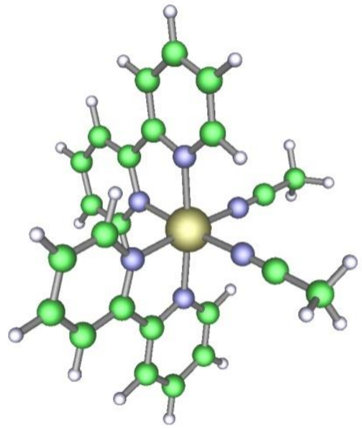

53
geometry

C	-2.80437659	2.28698751	-2.80473044
C	-2.17596090	2.91009995	-1.73383449
C	-1.26226436	2.19959848	-0.95481602
N	-0.97240528	0.89864739	-1.23179712
C	-1.58346148	0.30308813	-2.26997360
C	-2.50319003	0.95698491	-3.07746205
C	-0.71891969	4.09655736	0.63123607
C	-0.00673842	4.56065481	1.72975700
C	0.86721972	3.69391928	2.37855205
C	0.99200739	2.39536609	1.90421440
N	0.30083232	1.93693323	0.84588373
C	-0.55111047	2.77942620	0.20077748
C	0.08039792	-4.55788981	-1.71670711
C	-0.72378686	-4.08079160	-0.68924781
C	-0.57469391	-2.76639881	-0.24387017
N	0.34477688	-1.93789571	-0.80916286
C	1.12467598	-2.40900512	-1.79798851
C	1.02576750	-3.70657364	-2.28015084
C	-2.36336674	-2.87222348	1.54165341
C	-3.06639926	-2.24264904	2.56120927
C	-2.76173684	-0.92124925	2.86885458
C	-1.76538824	-0.28115795	2.14558024
N	-1.08169696	-0.88272385	1.15728465
C	-1.37281392	-2.17621318	0.84796933
Ru	0.44360200	-0.00138816	0.02270153
H	-3.51519448	2.83283518	-3.41380651
H	-2.39977001	3.94477999	-1.51144254
H	-1.31746763	-0.72945328	-2.45176199
H	-2.96779668	0.42813444	-3.90048216
H	-1.39985365	4.76086886	0.11605773
H	-0.13289951	5.58169437	2.07082227

H	1.44562199	4.01193742	3.23725113
H	1.66150381	1.68827579	2.37564346
H	-0.02968805	-5.57716756	-2.06803265
H	-1.46013353	-4.73355252	-0.23983821
H	1.84520862	-1.71258448	-2.20556846
H	1.67857059	-4.03573740	-3.07933616
H	-2.58881518	-3.90040078	1.29294738
H	-3.83659251	-2.77733340	3.10446107
H	-3.28217974	-0.38830153	3.65508623
H	-1.49593809	0.74494330	2.35664129
N	1.95376722	0.72000168	-1.18255526
C	2.79474442	1.16683719	-1.83004580
C	3.85749665	1.73043339	-2.64649955
H	3.43804438	2.21732722	-3.52940762
H	4.54362750	0.94584223	-2.97242489
H	4.42075420	2.47006517	-2.07350265
N	1.85044316	-0.74836676	1.33292720
C	2.64206891	-1.22332091	2.02127502
C	3.64490682	-1.82798654	2.88280516
H	3.47170659	-2.90330334	2.96271801
H	3.60281232	-1.39267421	3.88331684
H	4.64324277	-1.66506614	2.47120862

Table S1 X-ray Crystallographic data for complexes **1d**, **1e**, **2a** and **3d**.

	**1d**	**1e**	**2a**	**3d**
Formula	$C_{51.03}H_{45.58}F_{12}N_{14.49}O_{0.52}P_2Ru$	$C_{49}H_{41}F_{12}N_{15}OP_2Ru$	$C_{53}H_{50.5}F_{12}N_{17.5}P_2Ru$	$C_{34}H_{30}F_{12}N_{10}P_2Ru$
M_r / g mol^{-1}	1260.99	1246.98	1323.62	969.69
Temperature / K	150	150	150	150
Space group	P42bc	P42bc	P1	P21/c1
a / Å	22.5345(6)	22.4167(10)	12.4144(3)	9.3177(2)
b / Å	22.5345(6)	22.4167(10)	14.3194(3)	34.2603(9)
c / Å	20.6805(3)	20.6681(9)	17.5321(4)	11.9338(3)
α / °	90	90	92.0042(11)	90
β / °	90	90	98.6530(10)	93.351(1)
γ / °	90	90	108.1437(10)	90
V / Å^3	10501.6(5)	10385.9(8)	2916.82(11)	3803.08(16)
Dc / g cm^{-1}	1.595	1.595	1.507	1.694
Z	8	8	2	4
μ_{Mo} / mm^{-1}	0.457	0.462	3.501	0.598
2θmax	33.160	28.280	68.24	29.570
N_{ref}	19704	11575	10624	10666
R_1	0.0439 (14247)	0.0459 (8307)	0.0512 (9376)	0.0353 (8918)
wR_2	0.0977 (19704)	0.1087 (11575)	0.1550 (10624)	0.0832 (10666)
S	1.015	1.030	1.039	1.030

X-ray crystallography

Single crystal X-ray diffraction data were collected on a Bruker Apex Duo diffractometer equipped with a graphite monochromated Mo(K*α*) (**1d**, **1e** and **4d**) or a Bruker D8 Venture Cu(K*α*) (**2a**) radiation source and a cold stream of N_2 gas. Summarized crystal and refinement data are presented in Table S1. Preliminary scans were employed to assess crystal quality, lattice symmetry, ideal exposure time *etc*. prior to collecting a full sphere of diffraction intensity data using SMART operating software.[1] Intensities were then integrated from several series of exposures, merged and corrected for Lorentz and polarisation effects using SAINT software.[2] Solutions were generated by conventional heavy atom Patterson or direct methods and refined by full-matrix non-linear least squares on all F^2 data, using SHELXS-97 and SHELXL software respectively (as implemented in the SHELXTL suite of programs).[3] Empirical absorption corrections were applied based on multiple and symmetry-equivalent measurements using SADABS.[4] All structures were refined until convergence (max shift/esd < 0.01) and in each case, the final Fourier difference map showed no chemically sensible features. In some cases the structures contained disordered solvents which were restrained using DELU and SIMU in the least squares refinement. Structure **1d** contained substitutional disorder of acetonitrile and diethyl ether solvent molecules which was modeled using the PART instruction and **2a** contained a disordered acetonitrile molecule which as best modeled as half-occupancy and restrained using DELU, SIMU and ISOR for one of the carbon atoms.

Computational Details

The structures of *cis*- and *trans*-$[Ru(bpy)_2(NCMe)_2]^{2+}$ and *cis*- and *trans*-**3a** (benzyl substituents simplified to methyl) were optimized in the gas phase at the B3LYP[27] level if theory using the Stuttgart-Dresden relativistic small core potential[5] for Ru and 6-311G* basis sets[6] for all other atoms. Calculations were carried in using the NWChem 6.1 software package.[7]

Syntheses

Synthesis of [RuCl(p-cymene)(dmbpy)][PF$_6$].

[$RuCl_2$(*p*-cymene)]$_2$ (100.2 mg, 0.16 mmol) and 4,4'-dimethyl-2,2'-bipyridyl (120.5 mg, 0.65 mmol, 4 eq.) were suspended in 10 mL MeOH and the reaction mixture vigorously stirred at room temperature for 3 hours. After this time, an excess of NH_4PF_6 was added and the volume of the solution reduced by half *in vacuo*. An orange precipitate was observed to form which was filtered and washed with 10 mL Et_2O. Yield = 153.5 mg (78 %)

^{1}H NMR (500 MHz) CD_3CN δ_H 1.02 (s, 3H, CH(C*H*$_3$)), 1.04 (s, 3H, CH(C*H*$_3$)), 2.20 (s, 3H, *p*-cymene C*H*$_3$), 2.59 (s, 6H, 4-*Me*bpy), 2.64 (sp, $^3J_{HH}$ = 7.2 Hz, 1H, C*H*(CH$_3$)$_2$), 5.68 (d, $^3J_{HH}$ = 6.2 Hz, 2H, *p*-cymene Ar-C*H*), 5.89 (d, $^3J_{HH}$ = 6.2 Hz, 2H, *p*-cymene Ar-C*H*) 7.53 (d, $^3J_{HH}$ = 5.9 Hz, 2H, 4-Mebpy-*H*$_5$), 8.17 (s, 2H, 4-Mebpy-*H*$_3$), 9.14 (d, $^3J_{HH}$ = 6.0 Hz, 2H, 4-Mebpy-*H*$_6$). ^{13}C NMR (125.8 MHz) CD_3CN δ_C 18.5 (*C*H$_3$, *p*-cymene), 20.9 (*C*H$_3$, 4-*Me*bpy), 21.7 (*C*H$_3$, CH(*C*H$_3$)$_2$), 31.4 (CH, *C*H(CH$_3$)$_2$), 84.5 (*C*H, *p*-cymene Ar), 86.8 (*C*H, *p*-cymene Ar), 103.8 (*C*, *p*-cymene Ar-*C*(CH$_3$)), 105.0 (*C*, *p*-cymene Ar-*C*CH(CH$_3$)$_2$), 124.8 (*C*H, 4-Mebpy-*C*$_3$), 128.9 (*C*H, 4-Mebpy-*C*$_5$), 153.2 (*C*, 4-Mebpy-*C*$_4$), 154.8 (*C*, 4-Mebpy-*C*$_2$), 155.1 (*C*H, 4-Mebpy-*C*$_6$). HRMS-ESI calculated for $[RuClN_2C_{22}H_{26}]^+$ m/z = 455.082252, found m/z = 455.082790.

Synthesis of [RuCl(p-cymene)(phen)][PF$_6$].

[$RuCl_2$(*p*-cymene)]$_2$ (74.9 mg, 0.12 mmol) and 1,10-phenanthroline (88.1 mg, 0.49 mmol, 4 eq.) were suspended in 7 mL MeOH and the reaction mixture vigorously stirred at room temperature for 3 hours. After this time, an excess of NH_4PF_6 was added and the volume of the solution reduced by half *in vacuo*. An orange precipitate was observed to form – this was filtered and washed with 10 mL Et_2O. Yield = 109.2 mg (75 %)

^{1}H NMR (500 MHz) CD_3CN δ_H 1.01 (s, 3H, CH(C*H*$_3$)), 1.03 (s, 3H, CH(C*H*$_3$)), 2.19 (s, 3H, *p*-cymene C*H*$_3$), 2.71 (sp, $^3J_{HH}$ = 7.0 Hz, 1H, C*H*(CH$_3$)$_2$), 5.86 (d, $^3J_{HH}$ = 6.4 Hz, 2H, *p*-cymene Ar-C*H*), 6.05 (d, $^3J_{HH}$ = 6.4 Hz, 2H, *p*-cymene Ar-C*H*), 8.06 (dd, $^3J_{HH}$ = 5.3 Hz, 8.2 Hz, 2H, phen), 8.18 (s, 2H, phen), 8.78 (dd, $^4J_{HH}$ = 0.9 Hz, $^3J_{HH}$ = 8.3 Hz, 2H, phen), 9.69 (dd, $^4J_{HH}$ = 1.0 Hz, $^3J_{HH}$ = 5.3 Hz, 2H, phen). ^{13}C NMR (125.8 MHz) CD_3CN δ_C 18.4 (*C*H$_3$, *p*-cymene), 21.7 (*C*H$_3$, CH(*C*H$_3$)$_2$), 31.4 (CH, *C*H(CH$_3$)$_2$), 84.8 (*C*H, *p*-cymene Ar), 86.3 (*C*H, *p*-cymene Ar), 103.0 (*C*, *p*-cymene Ar-*C*(CH$_3$)), 106.0 (*C*, *p*-cymene Ar-*C*CH(CH$_3$)$_2$), 126.9 (*C*H, phen), 128.1 (*C*H, phen), 131.2 (*C*, phen), 139.4 (*C*H, phen), 146.3 (*C*, phen), 155.9 (*C*H, phen). HRMS-ESI calculated for $[RuClN_2C_{22}H_{22}]^+$ m/z = 451.050952, found m/z = 451.051132.

References

[1] SMART Diffracometer Control Software, Bruker Analytical X-ray Instruments Inc., Madison, WI, 1998.

[2] SAINT Integration Software, Siemens Analytical X-ray Instruments Inc., Madison, WI, 1994.

[3] SHELXTL Program System, Vers 5.1, Bruker Analytical X-ray Instruments Inc., Madison, WI, 1998.

[4] G. M. Sheldrick, SADABS: A Program for Absorption Correction with Siemens SMART System, University of Gottingen, Germany, 1996.

[5] P. J. Stephens, F. J. Devlin, C. F. Chabalowski, M. J. Frisch, *J. Phys. Chem.* **1994**, *98*, 11623-11627.

[6] D. Andrae, U. Haussermann, M. Dolg, H. Stoll, H. Preuss, *Theo. Chim. Acta* **1990**, *77*, 123-141

[7] R. Krishnan, J. S. Binkley, R. Seeger, J. A. Pople, *J. Chem. Phys.* **1980**, *72*, 650-654.

[8] M. Valiev, E. J. Bylaska, N. Govind, K. Kowalski, T. P. Straatsma, H. J. J. van Dam, D. Wang, J. Nieplocha, E. Apra, T. L. Windus, W. A. de Jong, *Comput. Phys. Commun.* **2010**, *181*, 1477.
